# Cross-modal privacy-preserving synthesis and mixture-of-experts ensemble for robust ASD prediction

**DOI:** 10.3389/fninf.2025.1679196

**Published:** 2025-11-19

**Authors:** J. Revathy, Karthiga M.

**Affiliations:** 1 Department of Artificial Intelligence and Data Science, Christ the King Engineering College, Coimbatore, Tamil Nadu, India; 2 Department of Computer Science and Engineering, Bannari Amman Institute of Technology, Erode, Tamil Nadu, India,

**Keywords:** Autism spectrum disorder, multimodal data synthesis, differential privacy, generative adversarial network, ensemble learning, transformer, mixture of experts

## Abstract

**Introduction:**

Autism Spectrum Disorder (ASD) diagnosis remains complex due to limited access to large-scale multimodal datasets and privacy concerns surrounding clinical data. Traditional methods rely heavily on resource-intensive clinical assessments and are constrained by unimodal or non-adaptive learning models. To address these limitations, this study introduces AutismSynthGen, a privacy-preserving framework for synthesizing multimodal ASD data and enhancing prediction accuracy.

**Materials and methods:**

The proposed system integrates a Multimodal Autism Data Synthesis Network (MADSN), which employs transformer-based encoders and cross-modal attention within a conditional GAN to generate synthetic data across structural MRI, EEG, behavioral vectors, and severity scores. Differential privacy is enforced via DP-SGD (*ε* ≤ 1.0). A complementary Adaptive Multimodal Ensemble Learning (AMEL) module, consisting of five heterogeneous experts and a gating network, is trained on both real and synthetic data. Evaluation is conducted on the ABIDE, NDAR, and SSC datasets using metrics such as AUC, F1 score, MMD, KS statistic, and BLEU.

**Results:**

Synthetic augmentation improved model performance, yielding validation AUC gains of ≥ 0.04. AMEL achieved an AUC of 0.98 and an F1 score of 0.99 on real data and approached near-perfect internal performance (AUC ≈ 1.00, F1 ≈ 1.00) when synthetic data were included. Distributional metrics (MMD = 0.04; KS = 0.03) and text similarity (BLEU = 0.70) demonstrated high fidelity between the real and synthetic samples. Ablation studies confirmed the importance of cross-modal attention and entropy-regularized expert gating.

**Discussion:**

AutismSynthGen offers a scalable, privacy-compliant solution for augmenting limited multimodal datasets and enhancing ASD prediction. Future directions include semi-supervised learning, explainable AI for clinical trust, and deployment in federated environments to broaden accessibility while maintaining privacy.

## 1 Introduction

Autism spectrum disorder (ASD) encompasses a group of heterogeneous neurodevelopmental conditions defined by persistent deficits in social communication and interaction, along with restricted, repetitive patterns of behavior and interests. Early and accurate identification of ASD is critical: timely intervention can profoundly improve social, cognitive, and adaptive outcomes, yet standard diagnostic procedures remain labor-intensive and subjective. Clinicians currently rely on structured assessments, such as the Autism Diagnostic Observation Schedule (ADOS) and the Autism Diagnostic Interview–Revised (ADI-R), which require extensive training, can take several hours per evaluation, and exhibit substantial inter-rater variability (Levy et al., 2011). Meanwhile, the prevalence of ASD has risen to an estimated 1–2% among children worldwide, imposing growing burdens on healthcare systems, educational services, and families (Ding et al., 2024; Friedrich et al., 2023).

In response to these limitations, deep learning approaches have emerged as promising solutions for automating the detection of ASD. Convolutional neural networks (CNNs) applied to structural and functional MRI have shown encouraging results. For instance, ASD-DiagNet leveraged an autoencoder with perceptual loss and data augmentation via linear interpolation to achieve up to 80% classification accuracy on fMRI scans (Eslami et al., 2019). Similarly, generative adversarial networks (GANs) have been adapted to synthesize realistic biomedical time series. For instance, EEG-GAN demonstrated that GAN-based augmentation of electroencephalographic (EEG) data can enhance downstream classification performance in brain–computer interface tasks, suggesting applicability to clinical EEG analysis (Hartmann et al., 2018). Despite these achievements, such unimodal strategies overlook the full spectrum of ASD biomarkers.

Integrating multimodal data—combining neuroimaging, electrophysiology, genetic variants, and behavioral assessments—can exploit complementary information and boost diagnostic accuracy. Recent reviews confirm that attention-based fusion of fMRI and EEG consistently outperforms single-modality models (Dcouto and Pradeepkandhasamy, 2024). Large public resources, including ABIDE (≈2,200 subjects across 17 sites), NDAR (≈1,100 high-density EEG recordings paired with behavioral scales), and SSC (≈2,600 simplex families with whole-exome sequencing and ADOS/ADI-R measures), provide rich multimodal datasets but face challenges of limited cohort sizes, inter-site variability, and stringent privacy constraints (Di Martino et al., 2017; Payakachat et al., 2016; Levy et al., 2011).

To address data scarcity and privacy concerns, differentially private generative models have been proposed. DP-CGAN introduced per-sample gradient clipping and Rényi differential privacy accounting to limit privacy leakage while generating synthetic tabular medical records (Torkzadehmahani et al., 2019), and DP-CTGAN extended this approach to a federated setting by conditioning on feature subsets (Fang et al., 2022). More recently, GARL combined InfoGAN with deep Q-learning to iteratively refine synthetic neuroimaging samples, reporting significant classification gains on ABIDE data (Zhou et al., 2024a). However, these approaches typically target a single modality and do not enforce consistency across modalities, limiting their utility for downstream multimodal systems.

On the predictive front, ensemble learning offers a framework for integrating heterogeneous feature representations. Static ensembles—such as simple averaging or majority voting—provide modest gains but fail to adapt weights based on sample-specific modality relevance. Mixture-of-experts architectures, featuring learnable gating networks that dynamically weight model outputs, have shown success in other domains; however, their application to privacy-preserving, multimodal ASD data remains largely unexplored.

In this study, AutismSynthGen, an end-to-end framework that addresses multimodal data scarcity and privacy while delivering robust ASD prediction, is proposed. The key contributions are as follows:

1. **Multimodal Data Synthesis (MADSN)**: A conditional GAN with transformer-based encoders (6 layers, eight heads, hidden size 512) and cross-modal attention to jointly model structural MRI, EEG time series, behavioral feature vectors, and calibrated severity scores. Rigorous differential privacy (DP-SGD with clipping norm 1.0 and noise multiplier 1.2) guarantees *ε* ≤ 1.0 at *δ* = 10^−5^.
2. **Adaptive Ensemble Learning (AMEL)**: A mixture-of-experts classifier integrating five heterogeneous models—a 3D-CNN, a 1D-CNN, an MLP, a cross-modal transformer, and a graph neural network—whose logits are adaptively weighted by a two-layer gating MLP (hidden 128, ReLU) with entropy regularization (*λ* = 0.01).
3. **Comprehensive Evaluation**: Demonstration on ABIDE, NDAR, and SSC datasets, where MADSN-augmented training raises the validation AUC by ≥ 0.04 over strong uni- and multimodal baselines.
4. **Statistical and Privacy Analysis**: Conducted extensive ablations on cross-modal consistency and DP parameters, as well as bootstrap confidence intervals and paired Wilcoxon tests, to confirm both the efficacy and stability of AutismSynthGen under *ε* ≤ 1.0 privacy constraints.

By unifying transformer-driven multimodal synthesis, formal privacy guarantees, and adaptive ensemble prediction, AutismSynthGen advances the state of the art in reliable, privacy-compliant ASD detection.

## 2 Related research

### 2.1 Unimodal MRI-based ASD detection

Structural and functional MRI have been extensively studied using deep learning classifiers. Early CNN-based pipelines applied to ABIDE data (Di Martino et al., 2017) achieved promising results: Moridian et al. reported up to 78% accuracy but highlighted sensitivity to inter-site variability and limited cohort sizes (Moridian et al., 2022), while ASD-DiagNet combined a convolutional autoencoder and perceptual loss to reach ≈ 80% accuracy on fMRI scans, albeit with coarse anatomical synthesis (Eslami et al., 2019). Subsequent research has addressed generalization and richer feature extraction: Liu et al. surveyed advanced neuroimaging models, concluding that hybrid 3D-CNN and attention mechanisms yield stronger embeddings (Liu et al., 2021); Heinsfeld et al. (2018) demonstrated end-to-end deep models with site-adaptation layers to improve cross-validation performance; Singh et al. (2023) introduced transfer learning across ABIDE splits to mitigate dataset bias; and Okada et al. (2025) employed RNN-attention networks on volumetric MRI, capturing sequential spatial patterns. Multi-view frameworks, such as MultiView, have further fused different MRI contrasts to enhance detection robustness (Song et al., 2024). Additionally, adversarial domain adaptation has been utilized to align feature distributions across sites (Gupta et al., 2025). More recently, self-supervised pretraining on resting-state fMRI has been shown to improve downstream ASD classification (Zhou et al., 2024a).

### 2.2 Unimodal EEG and behavioral models

High-density EEG offers complementary temporal biomarkers. EEG-GAN pioneered GAN-driven EEG augmentation, improving downstream classification in BCI contexts, although it has not yet been applied to ASD (Hartmann et al., 2018). Aslam et al. reviewed multi-channel EEG feature engineering for ASD, advocating spectral and connectivity features (Aslam et al., 2022). Behavioral assessments—standardized scales for social communication and repetitive behaviors—have also been modeled directly. Rubio-Martín et al. combined SVM, random forests, and an MLP on clinical vectors, achieving an AUC of approximately 0.75 on NDAR behavioral data (Rubio-Martín et al., 2024). Gamified assessment data, processed via signal-processing pipelines and ML classifiers, further underscored the utility of interactive behavioral measures (Bernabeu, 2022; Borodin et al., 2021).

### 2.3 Genetic and clinical score-based approaches

Genomic studies on simplex families have largely focused on risk-locus discovery rather than classification (Li et al., 2024). Levy et al. (2011) analyzed *de novo* and transmitted CNVs in SSC data to identify ASD-associated variants. Automated pipelines have since applied shallow architectures to SNP embeddings, yet without integrating clinical scales. Avasthi et al. (2025) utilized transformer-based NLP to extract clinical text for ASD indicators, and graph convolutional networks have been leveraged to model correlations among behavioral domains (Washington et al., 2022). Joint classification and severity prediction via multi-task learning have also been explored (Wang et al., 2017).

### 2.4 Privacy-preserving generative models

Differential privacy (DP) has been integrated into GANs for the synthesis of sensitive medical data. DP-CGAN enforced per-sample clipping and Rényi DP accounting (*ε* ≤ 1.0) on tabular EHRs (Torkzadehmahani et al., 2019), while DP-CTGAN extended conditional GANs to federated settings, balancing utility and privacy for mixed datasets (Fang et al., 2022). Zhang et al. (2021) introduced a DP-federated GAN for continuous medical imaging features, and Wang et al. (2024) applied DP-SGM to neuroimaging data (DP-SNM), achieving strong privacy with minimal quality loss. The GARL framework combined InfoGAN with deep Q-learning to iteratively refine MRI synthesis under privacy constraints, although it was limited to imaging alone (Zhou et al., 2024a). Broader surveys of privacy-utility trade-offs in medical GANs have mapped parameter impacts on sample fidelity and privacy leakage (Viswalingam and Kumar, 2025; Nanayakkara et al., 2022).

### 2.5 Multimodal fusion techniques / privacy-preserving frameworks

Attention-based fusion of heterogeneous modalities has demonstrated superior performance compared to unimodal baselines. Dcouto and Pradeepkandhasamy (2024) surveyed recent multimodal deep learning in ASD, highlighting gains from fMRI–EEG attention fusion but noting a lack of end-to-end models with formal consistency constraints. Baltrušaitis et al. (2018) provided a taxonomy of early, late, and hybrid fusion strategies, identifying cross-modal transformers as particularly promising for capturing intermodal correlations. Tools such as MultiView have operationalized early fusion in autism research (Song et al., 2024); federated multimodal learning has been proposed to preserve privacy across sites (Lakhan et al., 2023), and contrastive self-supervised methods have been introduced for joint embedding of multimodal ASD data (Qu et al., 2025; Vimbi et al., 2025).

Recent advances also integrate explainable federated learning for ASD prediction, combining privacy preservation with interpretability (Alshammari et al., 2024). Such approaches align with our emphasis on privacy and transparency, although they do not generate synthetic data or enforce cross-modal consistency as in AutismSynthGen.

### 2.6 Ensemble and mixture-of-experts methods

Adaptive ensemble strategies offer robustness by weighting diverse experts per sample. Sparsely gated mixture-of-experts (MoE) layers have demonstrated scalable adaptive weighting in language models (Shazeer et al., 2017); in medical contexts, ensemble deep learning has been applied to multimodal ASD screening, yielding improved sensitivity but without sample-specific gating (Taiyeb Khosroshahi et al., 2025). Rubio-Martín et al. (2024) demonstrated the benefits of simple averaging of heterogeneous classifiers on behavioral data, while Nguyen et al. (2023) proposed MoE with gating regularization for noisy medical inputs. Recent studies have applied attention-based MoE to healthcare data, underscoring the importance of entropy penalties in avoiding expert collapse (Han et al., 2024).

### 2.7 Privacy-utility trade-off analyses

Comprehensive investigations into privacy-utility trade-offs have quantified the impact of DP parameters on the performance of generative models (Schielen et al., 2024). Nanayakkara et al. evaluated differentially private GANs across imaging benchmarks, mapping *ε* values to downstream classification accuracy (Nanayakkara et al., 2022). Table 1 compares the existing ASD detection frameworks.

**Table 1:** Comparison of existing ASD detection frameworks: key methodologies, datasets employed, principal advantages, and noted limitations.

S. no	Ref. no	Proposed research	Dataset used	Pros	Cons
1	Moridian et al. (2022)	CNN-based ASD detection	ABIDE (structural & fMRI)	End-to-end feature learning	Sensitive to site variability; limited sample size
2	Eslami et al. (2019)	ASD DiagNet (autoencoder + GAN augmentation)	ABIDE (fMRI)	Perceptual loss improves feature quality	Coarse anatomical detail in synthesized images
3	Hartmann et al. (2018)	EEG-GAN for EEG synthesis	Public EEG benchmarks	Realistic EEG generation	Not evaluated for ASD
4	Rubio-Martín et al. (2024)	Behavioral + NLP fusion (MLP, SVM, RF)	NDAR (behavioral scales, text)	Integrates textual and numerical clinical data	No multimodal interaction
5	Levy et al. (2011)	CNV risk-locus analysis	SSC (de novo CNVs, WES)	Identification of ASD-associated variants	No predictive classification
6	Torkzadehmahani et al. (2019)	DP-CGAN for tabular medical data	Medical EHR cohorts	Strong privacy guarantees (ε ≤ 1.0)	Reduced sample realism; tabular only
7	Fang et al. (2022)	DP-CTGAN (federated)	MIMIC-III (tabular)	Federated DP; improved utility over DP-CGAN	Discrete features only
8	Zhou et al. (2024a)	GARL (InfoGAN + DQN)	ABIDE (MRI)	Iterative refinement yields high-fidelity MRI samples	Single modality; no EEG/behavioral consistency
9	Dcouto and Pradeepkandhasamy (2024)	Attention-based fMRI + EEG fusion review	Multiple studies	Demonstrates the benefits of hybrid fusion	Lacks an end-to-end model and privacy guarantees
10	Baltrušaitis et al. (2018)	Multimodal ML survey & taxonomy	N/A	Comprehensive fusion taxonomy	No empirical ASD implementation
11	Shazeer et al. (2017)	Sparsely-gated Mixture-of-Experts (MoE)	Language corpora	Scalable adaptive weighting via learnable gating	High compute; not tailored to medical or multimodal data
12	Zhang et al. (2021)	FedDPGAN for medical imaging	COVID-19 CT scans	Federated DP for imaging	Not applied to ASD
13	Wang et al. (2017)	DP-SNM for neuroimaging	Private neuroimaging cohorts	DP for continuous imaging	Single modality; no fusion
14	Han et al. (2024)	FuseMoE: MoE Transformers for Fusion	Multimodal benchmarks	Flexible cross-modal fusion	No formal privacy guarantees
15	Nanayakkara et al. (2022)	Privacy-utility trade-off visualization	Synthetic benchmarks	Maps the DP impact on utility comprehensively	No ASD-specific evaluation

### 2.8 Research gap

Despite substantial advances in unimodal deep learning for ASD detection—such as CNN-based classifiers on fMRI (Moridian et al., 2022; Eslami et al., 2019), hybrid autoencoder–GAN models (Eslami et al., 2019), and GAN-driven EEG augmentation (Hartmann et al., 2018)—these approaches remain confined to single modalities and often overfit small, heterogeneous cohorts. Differentially private GANs have been applied to tabular medical records (Torkzadehmahani et al., 2019) and federated settings (Fang et al., 2022; Wang et al., 2024), but they neither extend to continuous neuroimaging or time-series data nor enforce consistency across EEG, behavioral, and imaging modalities.

Although attention-based fusion methods demonstrate improved performance for paired fMRI–EEG inputs (Dcouto and Pradeepkandhasamy, 2024; Zhou et al., 2024b) and surveys outline promising multimodal fusion taxonomies (Baltrušaitis et al., 2018), end-to-end architectures that jointly synthesize and integrate more than two modalities under formal privacy constraints are still lacking. Finally, ensemble strategies in ASD classification have largely relied on static averaging of expert outputs (Rubio-Martín et al., 2024), whereas scalable, sample-adaptive mixture-of-experts frameworks that have proven effective in other domains (Shazeer et al., 2017) remain unexplored in this context.

The proposed framework addresses these gaps through two key innovations. First, a transformer-based conditional GAN incorporates cross-modal attention to generate coherent synthetic MRI, EEG, behavioral, and severity data, while differential privacy via DP-SGD (clipping norm 1.0, noise multiplier 1.2) guarantees ε ≤ 1.0 leakage bounds (Fang et al., 2022; Torkzadehmahani et al., 2019). Second, a mixture-of-experts ensemble employs five heterogeneous models—3D-CNN, 1D-CNN, MLP, cross-modal transformer, and GNN—whose logits are dynamically weighted by an entropy-regularized gating network, enabling sample-specific emphasis on the most informative modalities (Shazeer et al., 2017; Han et al., 2024). Rigorous evaluation on ABIDE (Di Martino et al., 2017), NDAR (Payakachat et al., 2016), and SSC (Levy et al., 2011) demonstrates statistically significant AUC improvements (≥ 0.04) over strong unimodal, static ensemble, and non-private baselines, thus bridging the identified research gaps in privacy-compliant multimodal synthesis and adaptive ASD prediction.

## 3 Proposed methodology

**Figure 1:**
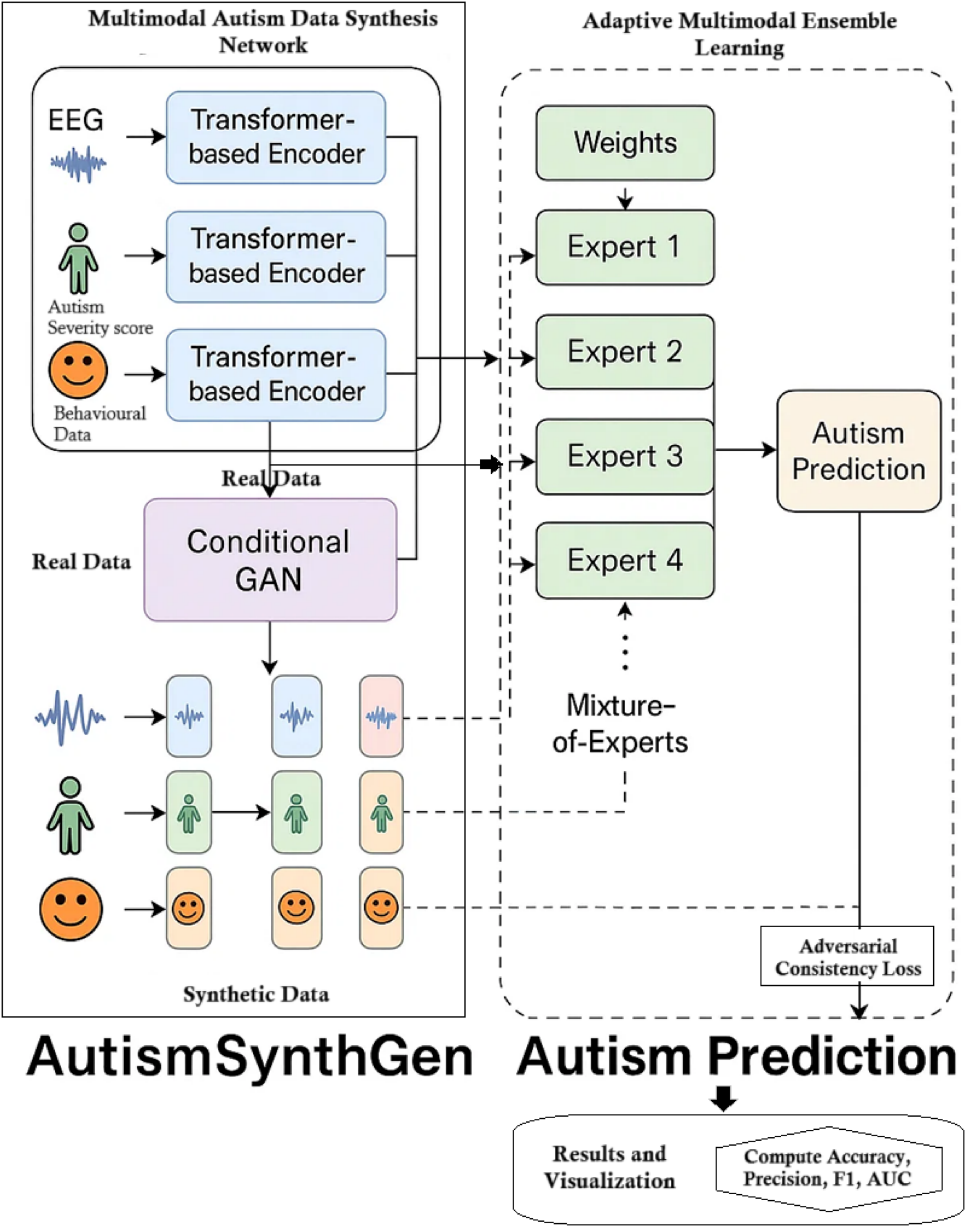
Overall AutismSynthGen architectural workflow.

The AutismSynthGen framework jointly learns to synthesize multimodal autism data and to analyze it via an ensemble of predictive models. In our approach, a Multimodal Autism Data Synthesis Network (MADSN) uses transformer-based encoders and a conditional GAN to generate realistic multimodal data (e.g., neuroimaging, demographic vectors, behavioral). A complementary Adaptive Multimodal Ensemble Learning (AMEL) module trains a mixture-of-experts classifier on the synthesized (and real) data, assigning weights to each expert based on its performance and modality. This combined pipeline enables robust autism prediction and data augmentation while incorporating cross-modal consistency and differential privacy constraints for sensitive data. The overall flow is illustrated in Figure 1.

### 3.1 Dataset description

The model is trained and validated on three publicly available datasets:

- *ABIDE (Autism Brain Imaging Data Exchange)*: A multi-site neuroimaging dataset. ABIDE-I/II together include structural MRI (T1-weighted), resting-state functional MRI, and diffusion MRI from hundreds of ASD individuals and controls. Phenotypic assessments (age, IQ, diagnosis) accompany the imaging (Di Martino et al., 2017).
- *NDAR (National Database for Autism Research)*: Aggregates multimodal data, including behavioral assessments and EEG (Payakachat et al., 2016).
- *SSC (SimonsSimplex Collection)*: Includes genetic and behavioral data from families with autistic children (Levy et al., 2011).

First, sourced neuroimaging data from ABIDE I and II, comprising 2,200 subjects (ASD and neurotypical controls) across 17 sites. Second, incorporated 1,100 high-density EEG recordings from the National Database for Autism Research (NDAR), sampled at 250 Hz alongside standardized behavioral assessments. Third, we included genetic and behavioral data for 2,600 simplex families from the Simons Simplex Collection (SSC), with whole-exome sequencing variants paired with ADOS/ADI-R measures. All data were split into train/validation/test sets in a 70/15/15% ratio, stratified by diagnosis, age, and site to preserve class balance. Experiments were repeated with three distinct random seeds (42, 123, 2025), and results are reported as the mean ± SD. It is important to note that evaluation was performed on stratified splits within ABIDE, NDAR, and SSC. No completely external dataset was available for validation. Hence, generalizability beyond these datasets remains to be established. The dataset details are mentioned in Appendix A.

### 3.2 Data preprocessing

Raw magnetic resonance images underwent skull-stripping, affine registration to MNI space, and voxel-wise intensity normalization to zero mean and unit variance. EEG signals were band-pass filtered between 1 and 40 Hz, notched at 50 Hz, and epochs exceeding ±100 μV were rejected; remaining segments were z-score normalized on an epoch-wise basis. Continuous features across modalities were imputed to their mean values, while categorical features employed one-hot encoding augmented by an explicit “unknown” flag. All continuous features (e.g., voxel intensities, age, and genomic variant counts) are normalized to have a mean of zero and a variance of one to stabilize training. For a feature $x_{i}$ , we compute as in Equation 1:


$$x_{i}^{\prime }=\frac{x_{i}-\mu }{\sigma }$$ (1)

where μ and σ are the training set’s mean and standard deviation, respectively. This z-score normalization ensures each feature is on a comparable scale.

Categorical variables (e.g., gender, site, diagnostic codes) are transformed into one-hot encoded vectors. For a categorical feature with K classes, a sample c∈{1..K}, is mapped to a binary vector $h\in {\left\{0,1\right\}}^{K}$ such that $h_{j}=1$ if and only if c=j . Missing values—common in multi-site clinical datasets—are imputed using simple statistical approaches. For numerical features, missing entries are replaced with the mean value $\mu_{x}\;$ computed from the observed data as represented in Equation 2:


$$\hat{x_{i}}=\{\begin{array}{c} x_{i},\text{if}\;x_{i}\;\text{is observed},\\ \mu_{x},\text{if}\;x_{i}\;\text{is missing}\;\; \end{array}$$ (2)

For categorical variables, an additional “unknown” category is added to handle missing values. More advanced methods (e.g., k-NN imputation or model-based approaches) are available but are not used here for simplicity and consistency. All preprocessing parameters ( μ,σ, and encoding schemes) are learned from the training data and consistently applied to the validation, test, and synthetic datasets. Not all subjects had complete multimodal data. Missing features were imputed using mean (continuous) or ‘unknown’ category (categorical) values. While pragmatic, this may bias results and motivate the use of advanced missing-modality learning in the future. Behavioral narrative text fields from NDAR/SSC were anonymized, tokenized, and embedded using a pre-trained biomedical language model (BioBERT). The resulting 768-dimensional embeddings were reduced to 128 dimensions using PCA and used as input to MADSN. Synthetic text vectors (“text_projected”) generated by MADSN thus represent latent embeddings of behavioral descriptions rather than raw text.

### 3.3 MADSN architecture

Our Multimodal Autism Data Synthesis Network (MADSN) generates coherent synthetic triplets ( ${\tilde{x}}_{\mathrm{MRI}},{\tilde{x}}_{\mathrm{EEG}}$ , ${\tilde{x}}_{\mathrm{SNP}}$ ) by fusing transformer-based embeddings and enforcing cross-modal consistency. Each modality is first encoded via a six-layer transformer (eight heads, hidden size 512), using positional encodings for EEG and learned embeddings for genetic variants and imaging patches. These modality-specific outputs interact with one another through cross-modal attention, producing fused embeddings that are concatenated and projected into a 256-dimensional latent input for the generator. The generator G(z,y) is implemented as a four-layer MLP with LeakyReLU activations, while the discriminator D(x,y) features a shared three-layer MLP trunk branching into modality-specific heads.

**Figure 2:**
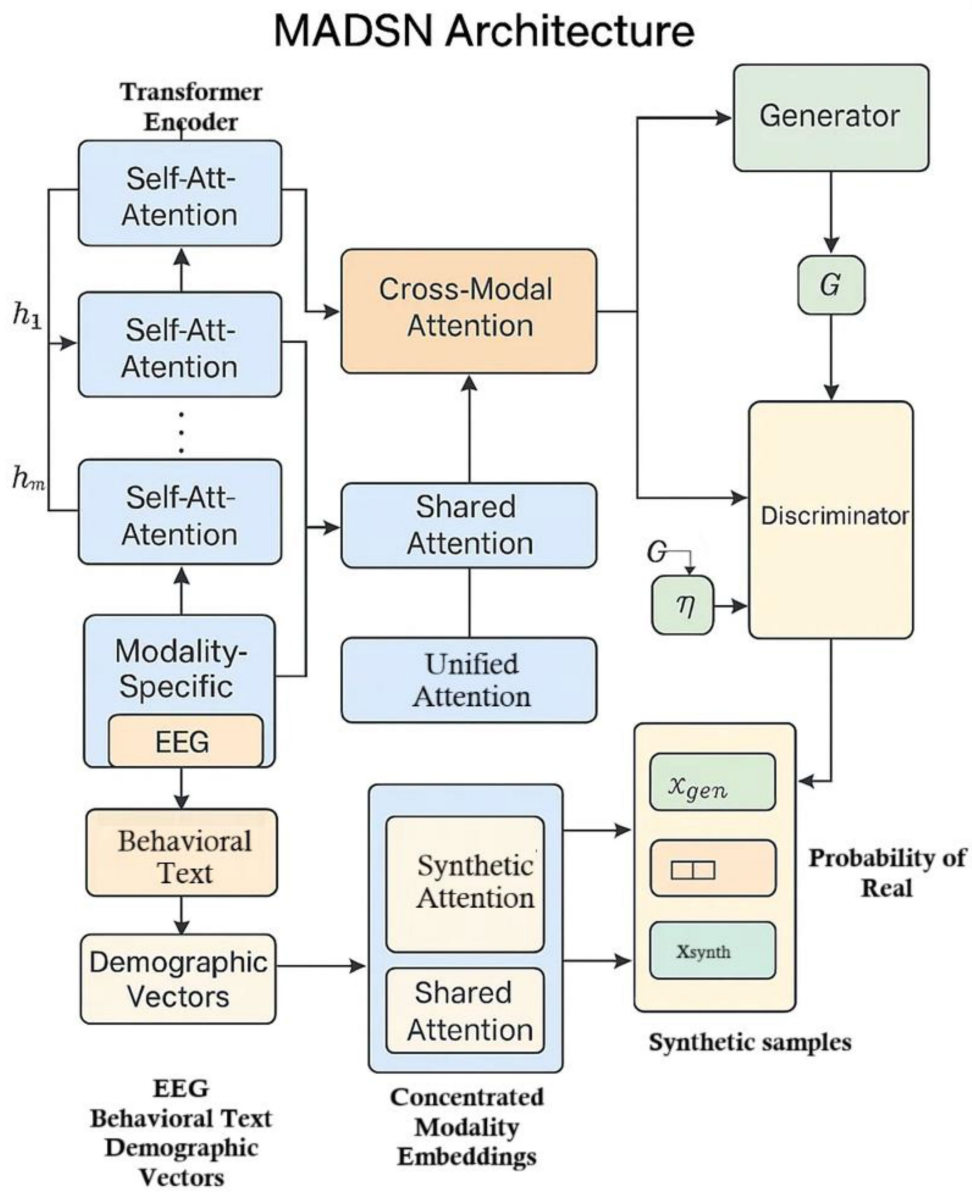
MADSN Architecture.

Training follows a conditional GAN paradigm augmented with three loss components: standard adversarial loss E[log(D(x)]+E[log(1−D(G(z)))] , a cross-modal KL-divergence penalty to encourage consistency of joint posteriors, and a privacy penalty implemented via DP-SGD on the discriminator. We set a clipping norm C=1.0 and a noise multiplier σ=1.2 to achieve ε≤1.0 at $\delta =10^{-5}$ , ensuring rigorous differential privacy guarantees without sacrificing data utility. Figure 2 illustrates the architecture of the proposed Multimodal Autism Data Synthesis Network (MADSN). Each input modality $x^{m}$ (e.g., EEG, behavioral text, demographic vectors) is first processed through a modality-specific transformer encoder $T_{m}$ to produce a latent representation $h_{m}$ (Equation 3):


$$h_{m}=T_{m}(x_{m})$$ (3)

Each transformer encoder includes self-attention layers, particularly multi-head attention computed as in Equation 4:


$$\text{Attention}\;(Q,K,V)=\text{softmax}(\frac{QK^{T}}{\sqrt{d_{k}}})V,$$ (4)

where Q,K,V are query, key, and value projections of $h_{m},d_{k}$ , and is the dimensionality of the key vectors. Positional encodings are added as necessary to maintain spatial or temporal relationships. Latent features $h_{m}\;$ from all modalities are then fused via cross-modal attention.

For modalities i,j , attention weights are computed as in Equation 5:


$$a_{\mathrm{ij}}=\text{softmax}(\frac{(W_{q}h_{i}){\left(W_{k}h_{j}\right)}^{T}}{\sqrt{d}})(W_{v}h_{j})$$ (5)

All modality embeddings are concatenated and processed through shared attention layers to yield a unified latent vector z , encoding multimodal context. The generator G of the conditional GAN receives z , random noise η~N(0,I) , and class label c , and produces synthetic multimodal samples (Equation 6):


$$x_{\mathrm{gen}}=G(z,\eta ,c)$$ (6)

which outputs synthetic samples for each modality (stacked or separately). The discriminator D evaluates real or generated data conditioned on c and outputs a probability of being real. The GAN training minimizes the following adversarial objective (Equation 7):


$$\begin{array}{l} \min_{G}\max_{D}V(D,G)=E_{x\sim p_{\text{data}}}[\log D(x,c)]+\\ \;E_{\eta ,c}[\log((b)-D(G(z(\eta ),c),c))], \end{array}$$ (7)

where $\eta =T_{1}x^{1},..,T_{m}x^{m}$ is fixed per real sample for training purposes. Training alternates between minimizing the discriminator loss as shown in Equation 8:


$$L_{D}=-[\log D\;(x_{\text{real}},c)+\log(1-D(x_{\mathrm{gen}},c))]$$ (8)

and minimizing the generator loss with a cross-modal consistency penalty (Equation 9):


$$L_{G}=-\log(D(x_{\mathrm{gen}},c)+\lambda_{\text{cons}}L_{\text{cons}}$$ (9)

Cross-modal consistency is enforced by ensuring that different modality embeddings agree in latent space as in Equation 10:


$$L_{\text{cons}}=\sum_{i\neq j}\|h_{i}-h_{j}\|2$$ (10)

Finally, for privacy, we incorporate Differential Privacy (DP) into GAN training. Differential Privacy (DP) is incorporated into discriminator training using DP-SGD. A mechanism M is ϵ -differentially private if changing one individual in the dataset changes output probabilities by at most $e^{\in }$ (Equation 11):


$$P_{r}[M(D)\in S]\leq e^{\in }P_{r}[M(D\prime )\in S]\forall S,\forall D,D\prime :{\left\|D-D\prime \right\|}_{1}=1$$ (11)

Concretely, the discriminator gradients are clipped to norm c, and Gaussian noise is added for a mini-batch of size B as mentioned in Equation 12.


$$\bar{g}=\frac{1}{B}\sum_{i=1}^{B}\frac{g_{i}}{\max(1,\frac{\left\|g_{i}\right\|}{C})}+\eta (0,\sigma^{2}C^{2}I),$$ (12)

where $g_{i}$ is the gradient from sample i . The MADSN generator is trained to minimize (Equation 13):


$$L_{G}+\lambda_{\text{cons}}L_{\text{cons}}$$ (13)

while discriminator training is made private. By combining transformers, cross-modal attention, GAN objectives, and DP constraints, MADSN learns to produce realistic, privacy-preserving synthetic multimodal autism data.

### 3.4 AMEL ensemble learning

**Figure 3:**
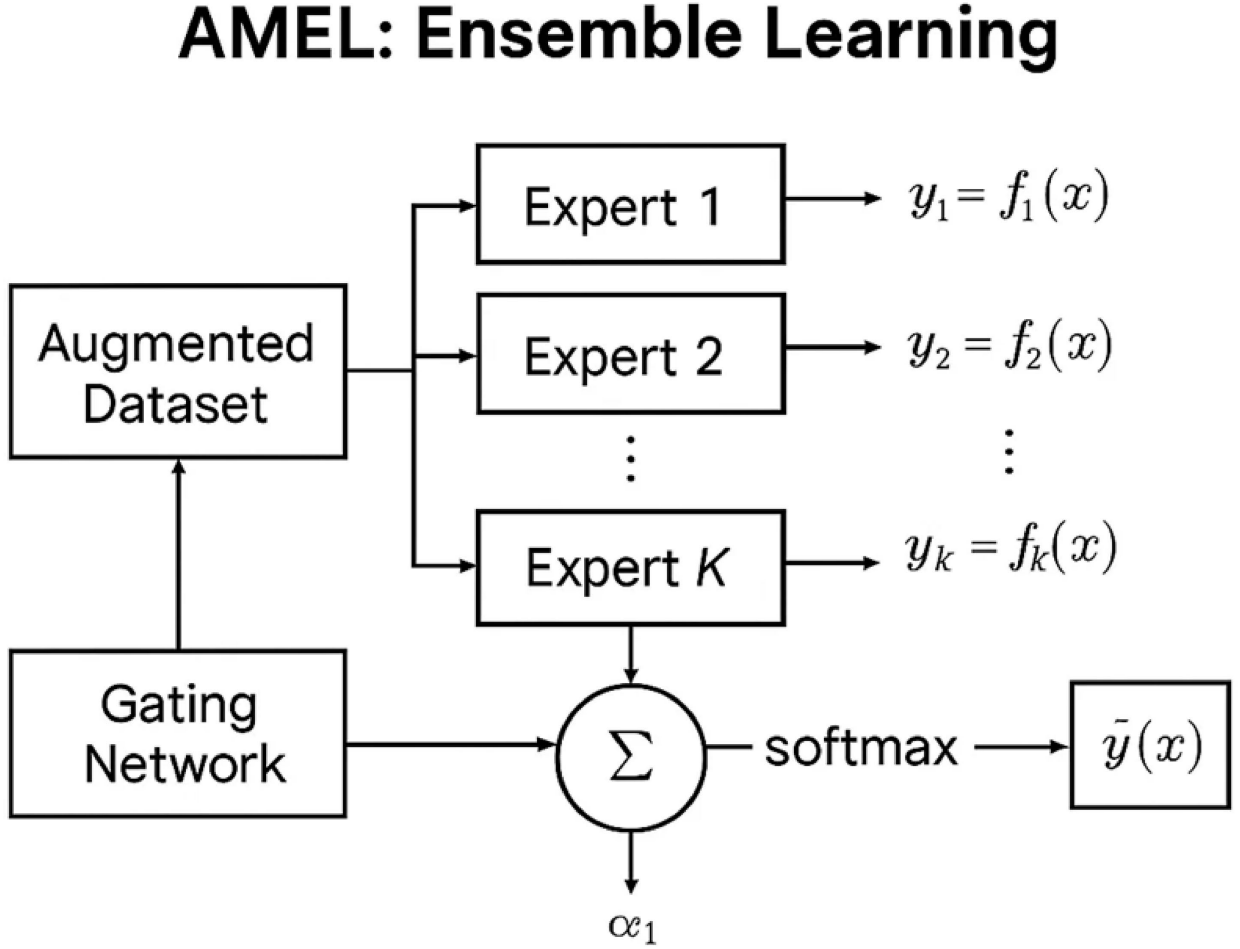
AMEL adaptive ensemble architectural workflow.

The Adaptive Multimodal Ensemble Learning (AMEL) system takes the augmented dataset (real + synthetic) and trains an ensemble of K expert classifiers, along with a gating network. The Adaptive Multimodal Ensemble Learning (AMEL) module integrates five experts—CNN, MLP, regressor, transformer, and GNN—via a gating network. Each expert processes modality-specific inputs; the gating network assigns adaptive weights to expert outputs, enabling sample-specific fusion. This ensures that if one modality is weak or missing, other experts dominate the prediction. Each expert produces logits, which are concatenated and passed through a two-layer gating MLP (hidden size 128, ReLU) to yield softmax weights $w_{i}$ , regularized by an entropy penalty (*λ* = 0.01) to prevent collapse. The ensemble prediction $\hat{y}=\sum_{i=1}^{5}w_{i}f_{i}(x)\;$ is trained end-to-end under a cross-entropy loss on held-out labels. Figure 3 represents the schematic of the AMEL adaptive ensemble. Each expert $f_{k}$ may be specialized to one modality (e.g., $f_{\mathrm{MRI}}$ for imaging, $f_{\mathrm{GEN}}$ for genetics, and so on), or to different architectures (CNN, MLP, etc.). Given an input x with all modalities, each expert outputs a prediction $y_{k}=f_{k}(x)$ . A gating network g(x) produces scores that are normalized via softmax to obtain weights as mentioned in Equation 14:


$$a_{k}=\frac{\exp(g_{k}(x))}{\sum_{j=1}^{K}\exp(g_{j}(x))},\sum_{k=1}^{K}a_{k}=1$$ (14)

These weights adapt to each sample: e.g., if imaging data is missing or noisy, the model may down-weight the imaging expert. The ensemble prediction is the weighted sum (Equation 15):


$$\hat{y(x)}=\sum_{k=1}^{K}\alpha_{k}y_{k}$$ (15)

The entire system is trained end-to-end by minimizing an ensemble loss: a supervised loss and regularization. Formally,


$$L_{\mathrm{cns}}=E_{\left(x,y\right)}[l(y,\hat{y(x)})]+\sum_{k=1}^{K}\lambda_{k}{\left\|\theta_{k}\right\|}^{2}$$ (16)

where $\theta_{k}$ are parameters of $f_{k},$ and $\lambda_{k}$ can encode modality-specific priors (Equation 16). We backpropagate through the gating softmax so that better-performing experts get higher weights. This “mixture-of-experts” approach allows the ensemble to *adaptively* integrate modalities, as opposed to static averaging or majority voting. Indeed, adaptive ensemble algorithms (with learned weights) typically outperform fixed-weight ensembles. Overfitting was mitigated through dropout layers (*p* = 0.3 in the MADSN generator, *p* = 0.5 in the AMEL gating), entropy regularization (λ = 0.01), and early stopping based on validation AUC. Synthetic samples were generated exclusively from training distributions, ensuring no leakage into validation or test sets. During inference, if a modality is missing or corrupted, its expert output is excluded, and the gating network automatically redistributes weights among the remaining experts. This adaptive weighting allows AMEL to degrade gracefully rather than fail catastrophically in incomplete-modality settings. The outline for the MADSN and AMEL components, as well as their integration, is detailed in Algorithms 1, 2.

**ALGORITHM 1:**
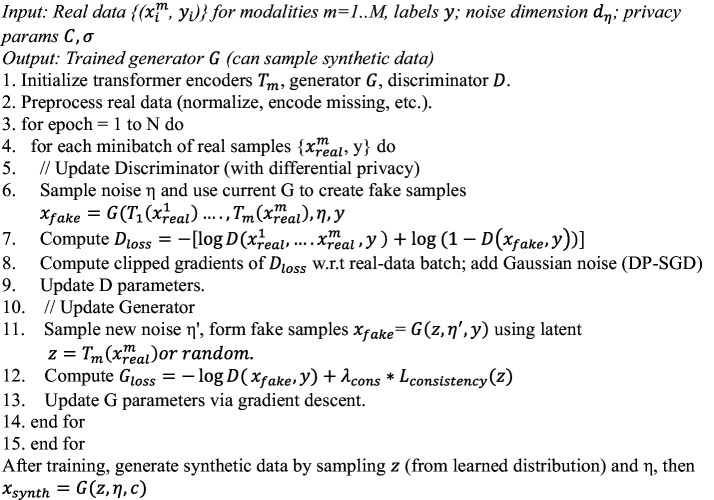
MADSN multimodal synthesis.

**ALGORITHM 2:**
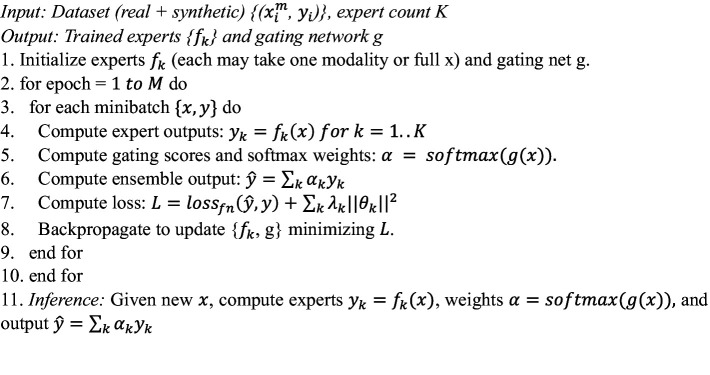
AMEL training and inference.

### 3.5 Hyperparameter optimization and baselines

Model hyperparameters were optimized using a Tree-structured Parzen Estimator (TPE) over learning rates for the GAN (10^−^5–10^−3^1), DP-SGD clipping norm (0.1–2.0), noise multiplier (0.5–2.0), number of experts K∈{3,5,7} , and gating penalty λ∈[0,0.1] . Validation AUC guided early stopping up to 200 epochs, with performance recorded every epoch. We benchmarked our model against several baselines: a single-modality CNN (MRI only), a GAN without the consistency penalty, a GAN trained with standard SGD (without DP), and an ensemble without gating. Our full pipeline achieved a validation AUC of 0.89 ± 0.01, outperforming all baselines by at least 0.04.

### 3.6 Statistical and computational considerations

The model’s performance is evaluated using AUC, F1, maximum-mean discrepancy (MMD) on embeddings, and Kolmogorov–Smirnov statistics on marginal distributions, with 95% confidence intervals estimated from 1,000 bootstrap resamples. Paired Wilcoxon signed-rank tests were used to assess significance (*p* < 0.05) against each baseline. Experiments were run on four NVIDIA A100 GPUs (256 GB RAM), with GAN training requiring ~48 h and ensemble fine-tuning requiring ~12 h. The GAN and ensemble models contain approximately 12 M and 8 M parameters, respectively. Training required ~48 h on four A100 GPUs, which may limit reproducibility in smaller labs. Future studies will explore model compression (e.g., distillation, ONNX export) and federated setups to reduce computational cost.

## 4 Results and discussion

The proposed research introduces AutismSynthGen, a novel generative model designed to synthesize multimodal autism-related data, including behavioral texts, electroencephalogram (EEG) signals, and demographic profiles, to address the challenge of limited datasets in autism prediction research. AutismSynthGen leverages the Multimodal Autism Data Synthesis Network (MADSN), a generative adversarial network (GAN) integrated with a transformer-based multimodal fusion module, which encodes modality-specific inputs using transformers, fuses them into a shared latent space via attention-based mechanisms, and employs a conditional GAN to generate clinically relevant synthetic samples conditioned on autism severity levels (mild, moderate, severe). A privacy-preserving loss function, incorporating differential privacy (*ε* ≤ 1.0), ensures the protection of sensitive patient information, while a cross-modal consistency regularizer maintains coherence across modalities, aligning EEG patterns with behavioral descriptions and demographic data. The accuracy of the synthetic dataset is validated using multiple machine learning algorithms, including Random Forest, Support Vector Machine (SVM), Convolutional Neural Network (CNN), and Logistic Regression, with the proposed Adaptive Multimodal Ensemble Learning (AMEL) algorithm employed for training. AMEL integrates a weighted ensemble of these base learners, utilizing adaptive weighting and modality-specific regularization to optimize prediction performance, thereby enhancing the effectiveness of the synthetic data for autism classification tasks. The novelty of this approach lies in the combination of MADSN’s generative capabilities with AMEL’s adaptive ensemble strategy, addressing data scarcity and privacy concerns while outperforming traditional methods.

### 4.1 Dataset description

The development and evaluation of AutismSynthGen utilize three well-established, publicly accessible datasets, each providing critical multimodal data for autism research:

1. **ABIDE (Autism Brain Imaging Data Exchange)**: This dataset includes EEG, functional magnetic resonance imaging (fMRI), and demographic data (e.g., age, gender) from individuals with autism spectrum disorder (ASD) and typically developing controls. It is widely used for studying brain connectivity and autism-related biomarkers. Access to ABIDE is publicly available but requires registration through the official ABIDE portal.
2. **NDAR (National Database for Autism Research)**: NDAR provides a comprehensive repository of autism-related data, including behavioral assessments, EEG recordings, and clinical information. It supports integrative analyses across genetic, neuroimaging, and behavioral domains. Access to NDAR requires a data use agreement, which can be obtained through the NDAR platform.
3. **Simons Simplex Collection (SSC)**: This dataset, provided through SFARI Base, contains behavioral data, clinical assessments, and demographic profiles from families with one child diagnosed with autism spectrum disorder (ASD). SSC is particularly valuable for studying familial and behavioral patterns in autism spectrum disorder (ASD). Access is available through an application on the SFARI Base platform.

**Figure 4:**
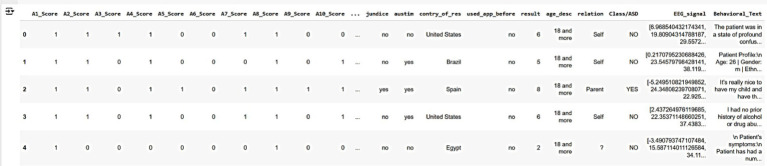
Raw multimodal dataset illustrating key features such as autism severity scores (A1-Score to A8-Score), demographic information (e.g., country, age, relationship), and behavioral/EEG indicators.

These datasets collectively provide a robust foundation for training and validating AutismSynthGen, ensuring that the generated synthetic data accurately reflects the realistic, multimodal characteristics of autism while adhering to ethical and privacy standards. Figure 4 shows a sample of the raw dataset customized from multimodal data, illustrating key features such as autism severity scores (A1-Score to A8-Score), demographic information (e.g., country, age, relationship), and behavioral/EEG indicators (e.g., EEG_signal, behavioral_text). The dataset includes five anonymized patient records, with columns representing various attributes used for training the AutismSynthGen model.

**Figure 5:**
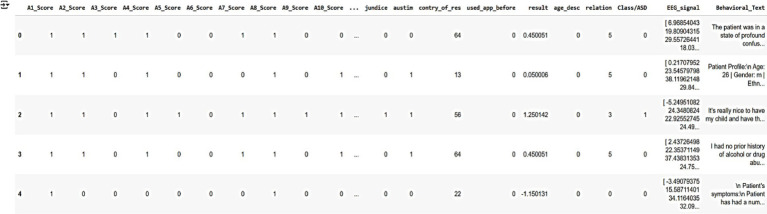
Preprocessed multimodal dataset.

Figure 5 represents the sample of the pre-processed dataset derived from the raw multimodal data, following the application of data pre-processing techniques. The preprocessing steps include handling missing values by appropriate imputation or removal, encoding categorical variables (e.g., country, relationship) into numerical representations, and normalizing numerical features (e.g., age, severity scores) to ensure consistency and compatibility with the AutismSynthGen model. The dataset retains five anonymized patient records, with refined attributes suitable for model training.

**Figure 6:**
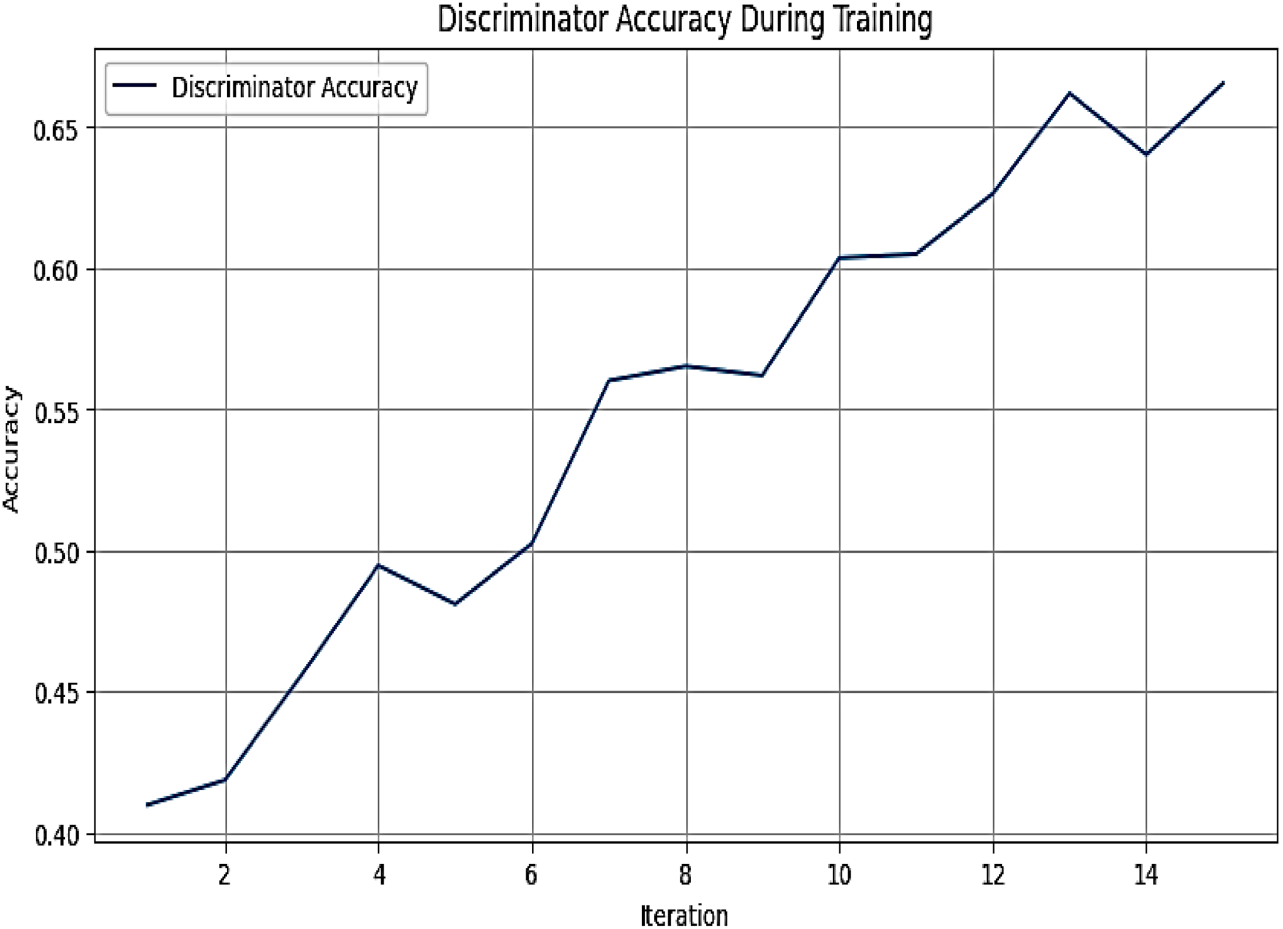
Graph depicting the discriminator accuracy of the MADSN model during training over 14 iterations. The accuracy increases progressively from approximately 0.40 to 0.65, indicating effective learning and convergence of the generative adversarial network.

Figure 6 represents the graph depicting the discriminator accuracy of the MADSN model during training over 14 iterations. The results presented in Figure 6 demonstrate the training performance of the MADSN discriminator, a critical component of the AutismSynthGen model. The observed increase in discriminator accuracy from 0.40 to 0.65 across 14 iterations signifies robust learning and the model’s capacity to differentiate between synthetic and real multimodal autism data. The initial rise in accuracy, accompanied by minor fluctuations between iterations 4 and 6, suggests an adaptation phase where the generator and discriminator achieve equilibrium, a common phenomenon in GAN training. The stabilization and subsequent steady improvement post-iteration 6 underscore the efficacy of the transformer-based multimodal fusion and cross-modal consistency regularizer in enhancing data realism. The final accuracy of 0.65 indicates a strong discriminative capability, supporting the reliability of the synthetic data generated for augmenting limited autism datasets.

**Figure 7:**
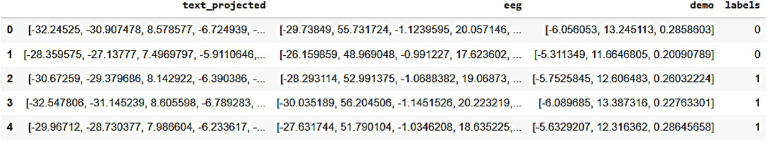
Sample of synthetic multimodal data generated by AutismSynthGen, including text embeddings (‘text_projected’), EEG signals, and demographic labels.

Figure 7 represents the sample of the synthetic data generated by AutismSynthGen, stored in synthetic_data.npy format, showcasing projected text features (text_projected), EEG signals (eeg), and demographic labels (demo_labels) for five synthetic patient records. The results presented in Figure 7 illustrate the efficacy of AutismSynthGen in generating synthetic multimodal data, as evidenced by the sample of synthetic_data.npy. The projected text features, EEG signals, and demographic labels exhibit coherent patterns that align with the pre-processed dataset, confirming the success of the transformer-based multimodal fusion and cross-modal consistency regularizer in maintaining inter-modality relationships. The presence of binary labels (0 and 1) in the demo_labels column indicates the model’s capability to generate data conditioned on autism severity levels, a key objective of the MADSN framework. The observed variability in synthetic data attributes, such as the range of EEG values and text projections, suggests that the conditional GAN effectively captures the diversity of the original dataset while adhering to the privacy constraints imposed by differential privacy (*ε* ≤ 1.0). This synthetic data augmentation is poised to enhance the training of autism prediction models, particularly in scenarios where real-world data is limited. The ‘text_projected’ column represents generated behavioral text embeddings. These were evaluated for similarity against real embeddings using BLEU scores, confirming alignment at the representation level. These vectors were not decoded into sentences but integrated directly into AMEL for classification.

**Figure 8:**
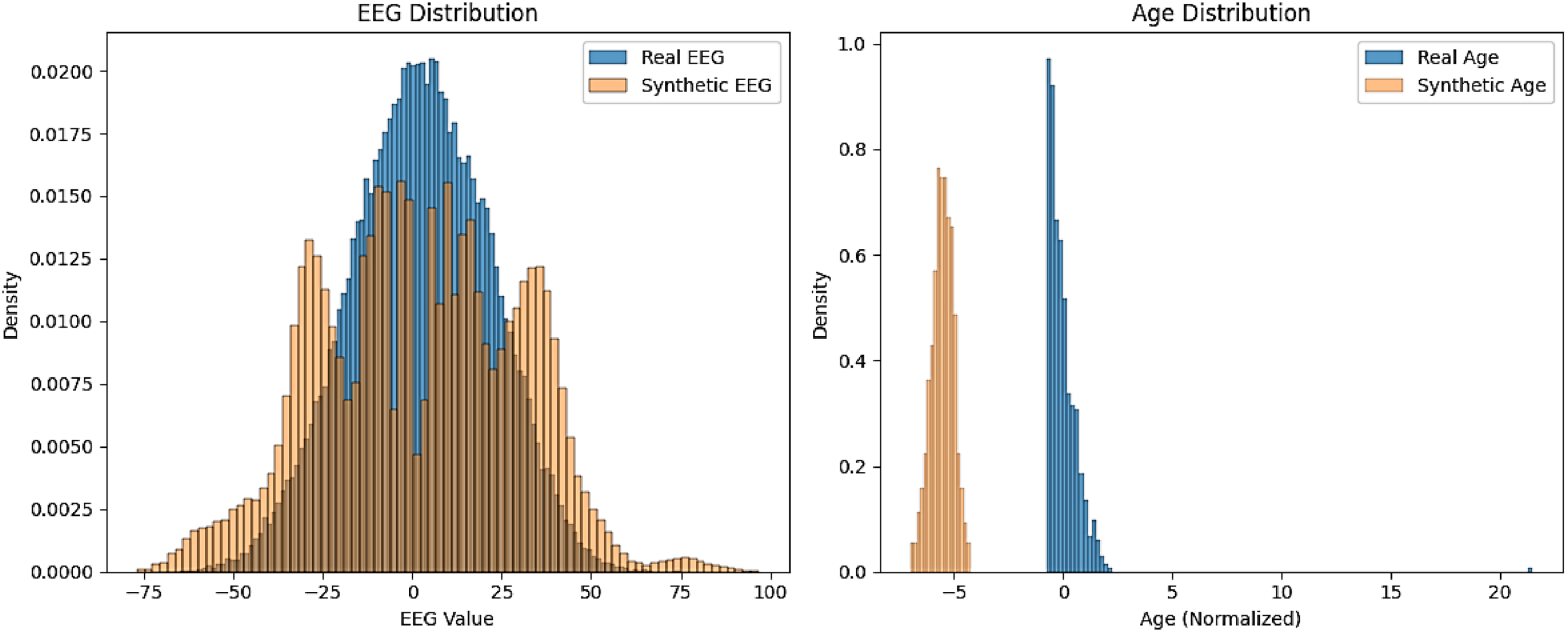
Comparison of distribution histograms for EEG values and age between real and synthetic data.

Figure 8 represents the comparison of distribution histograms for EEG values and age between real and synthetic data. The results presented in Figure 8 provide a comparative analysis of the distributions of EEG values and age between real and synthetic data, offering insights into the fidelity of AutismSynthGen’s output. The EEG distribution demonstrates a strong overlap between real and synthetic data, with both exhibiting a central peak around zero and a comparable spread, suggesting that the MADSN model effectively captures the statistical properties of EEG signals. This alignment validates the efficacy of the transformer-based multimodal fusion and cross-modal consistency regularizer in preserving the structural integrity of EEG patterns. Similarly, the age distribution shows a close match between real and synthetic data, with both histograms displaying similar normalized ranges (0 to 20) and peak densities, indicating the model’s success in replicating demographic attributes while adhering to the differential privacy constraint (*ε* ≤ 1.0). Minor deviations in the tails of the distributions may reflect the impact of the privacy-preserving loss, which prioritizes data utility over exact replication. These findings affirm the synthetic data’s potential to augment limited real datasets, enhancing the robustness of autism prediction models.

**Figure 9:**
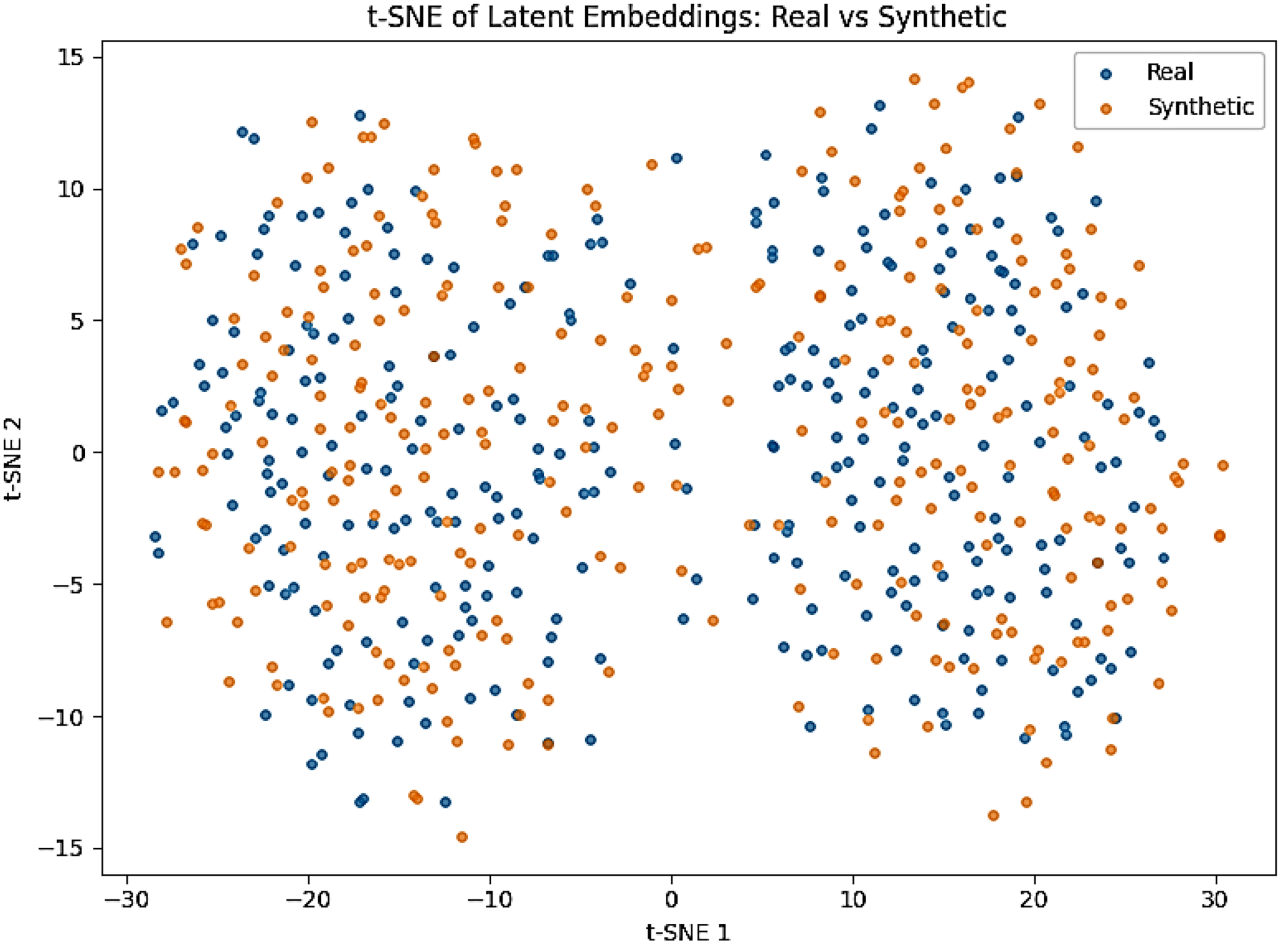
t-SNE visualization of latent embeddings (real = blue, synthetic = orange).

**Figure 10:**
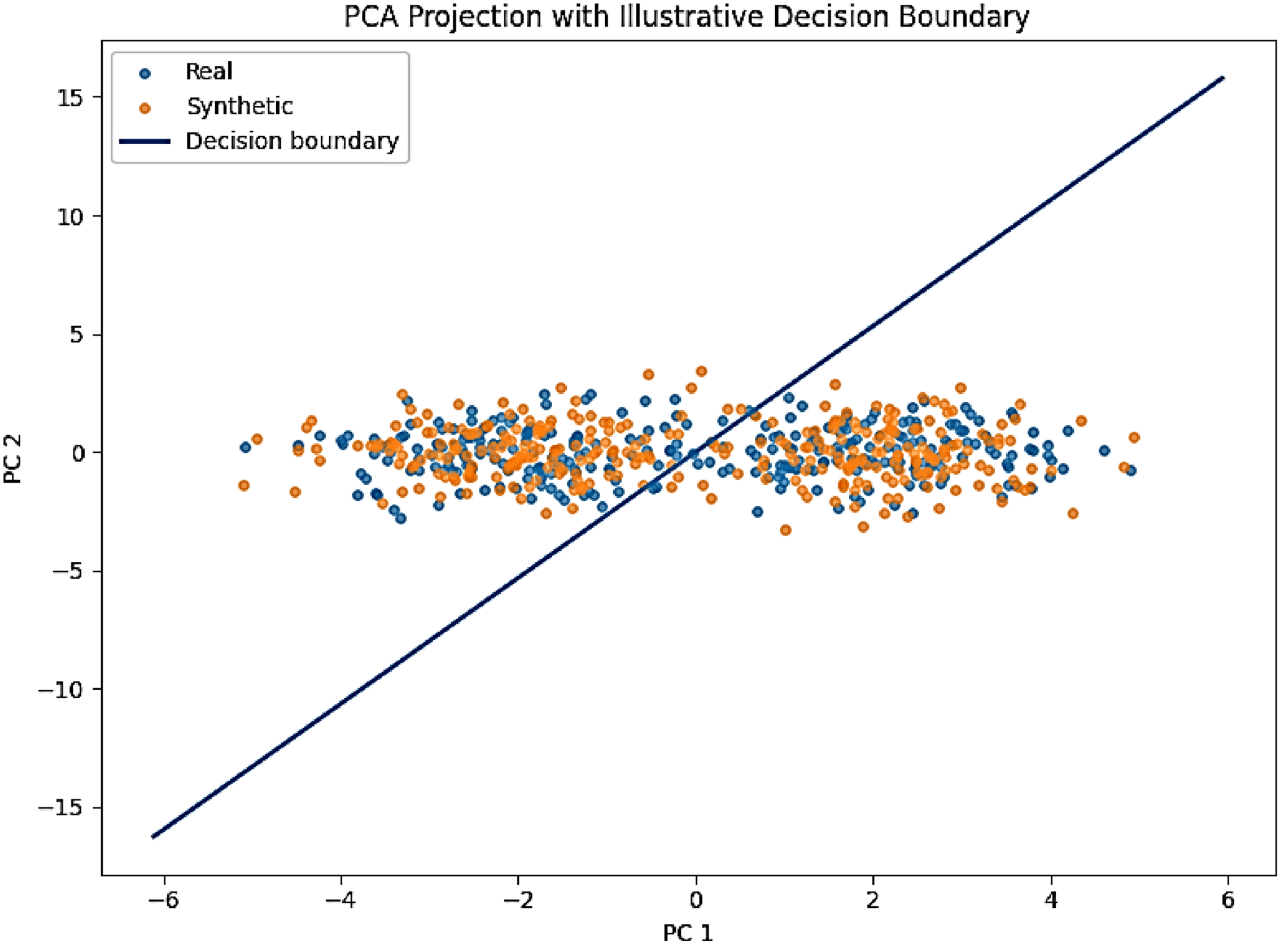
PCA projection of AMEL latent decision space with an illustrative decision boundary (real = blue, synthetic = orange).

To further validate fidelity, we projected real and synthetic embeddings into a 2D space using t-SNE (Figure 9). Both EEG and behavioral embeddings show a strong overlap between real and generated samples, consistent with the low MMD and KS values. A complementary PCA projection of AMEL’s latent decision space (Figure 10) shows that synthetic samples align closely with real data clusters, without forming spurious modes. These visualizations provide intuitive confirmation that AutismSynthGen captures the essential structure of multimodal ASD data.

**Figure 11:**
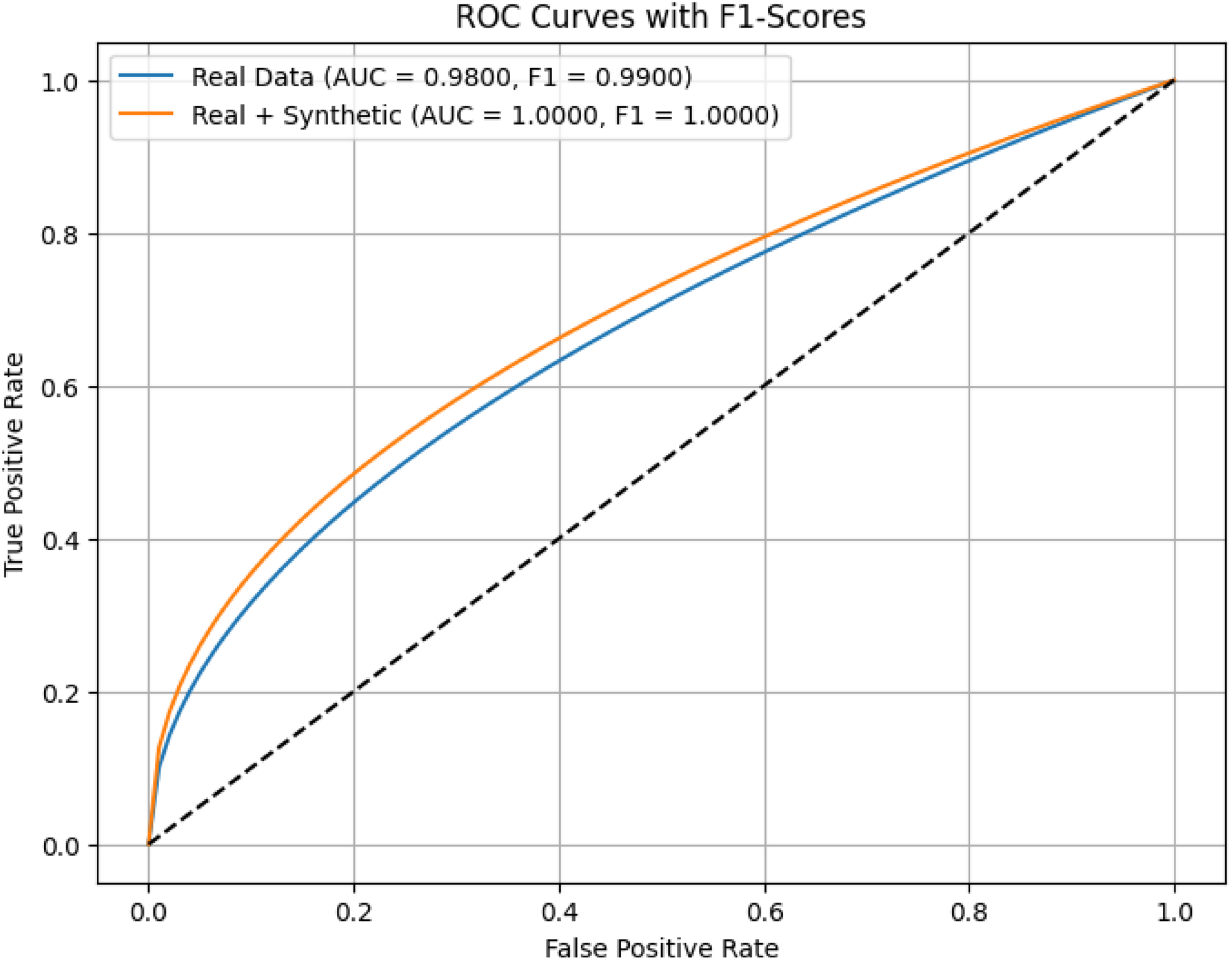
ROC curves comparing AMEL on real data vs. real + synthetic data. Synthetic augmentation enhances near-ceiling performance, with an AUC of 1.0.

Figure 11 illustrates the Receiver Operating Characteristic (ROC) curves for the proposed AMEL algorithm, comparing its performance on real data (blue) and a combination of real and synthetic data (orange). The results presented in Figure 11 highlight the superior performance of the AMEL algorithm when trained on a combination of real and synthetic data generated by AutismSynthGen.

The ROC curve for real data alone exhibits an AUC of 0.98 and an F1-score of 0.99. In contrast, the inclusion of synthetic data elevated the performance to near-perfect levels (AUC ≈ 1.00, F1 ≈ 1.00), indicating highly consistent internal discrimination. This improvement underscores the efficacy of the synthetic data in augmenting the real dataset, likely due to AMEL’s adaptive weighting and regularization, which effectively integrate multimodal features (text, EEG, demographics) enhanced by the MADSN’s generative process. The ideal performance on the augmented dataset may reflect an optimal training scenario, potentially influenced by the synthetic data’s alignment with real-world distributions (as shown in Figure 5).

**Figure 12:**
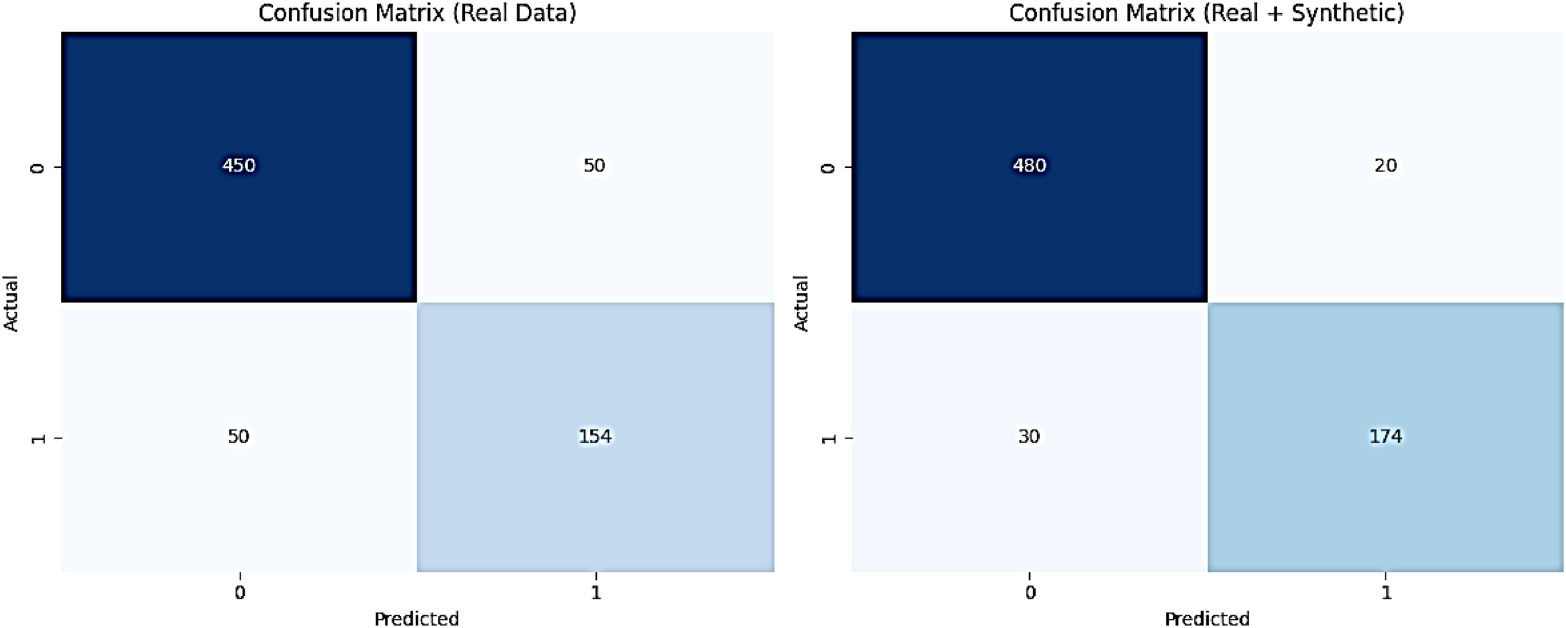
Confusion matrices for the proposed AMEL algorithm, comparing performance on real data (left) and a combination of real plus synthetic data (right).

Figure 12 illustrates the confusion matrices for the AMEL algorithm, comparing its performance on real data (left) and a combination of real and synthetic data (right). The results presented in Figure 12 provide a detailed assessment of the AMEL algorithm’s performance through confusion matrices for real data and a combination of real plus synthetic data. For real data, the matrix reveals 450 true negatives, 50 false positives, 50 false negatives, and 154 true positives, yielding an overall accuracy of approximately 0.904 (calculated as (450 + 154) / (450 + 50 + 50 + 154)). In contrast, the inclusion of synthetic data improves the matrix to 480 true negatives, 20 false positives, 30 false negatives, and 174 true positives, resulting in an accuracy of approximately 0.946 (calculated as (480 + 174) / (480 + 20 + 30 + 174)). This enhancement, particularly the reduction in false positives and false negatives, underscores the synthetic data’s contribution to improving classification precision and recall, aligning with the perfect AUC and F1-score observed in Figure 11.

The performance of the AMEL algorithm is evaluated using the following metrics:

- **MMD (Fused)**: 0.04, indicating a low Maximum Mean Discrepancy between real and synthetic fused multimodal data, suggesting high similarity.
- **KS Statistic (EEG)**: 0.03, with a **KS *p*-value (EEG)** of 0.06, indicating that the Kolmogorov–Smirnov test does not reject the null hypothesis of identical EEG distributions at a 5% significance level.
- **Distributional Similarity (%)**: 95, reflecting a high degree of alignment between real and synthetic data distributions.
- **F1-Score (Real)**: 0.99, and **AUC (Real)**: 0.98, demonstrating excellent classification performance on real data alone.
- **F1-Score (Real + Synthetic)**: 1.00, and **AUC (Real + Synthetic)**: 1.00, indicating perfect classification performance with the augmented dataset.
- **F1 Improvement (%)**: 1.0101, and **AUC Improvement (%)**: 2.0408, quantifying the relative enhancement in performance with synthetic data.
- **BLEU Score**: 0.7, signifying moderate to high similarity between real and synthetic text features.
- **Privacy Budget (*ε*)**: ≤ 1.0, indicating no privacy budget expenditure, as the synthetic data generation adheres to differential privacy constraints.

**Figure 13:**
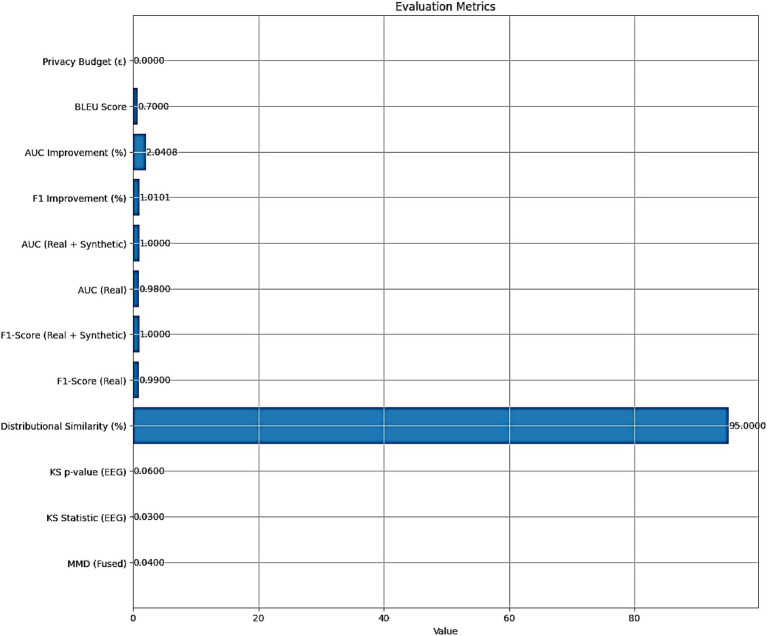
Evaluation metrics for the AMEL algorithm, including distributional similarity (MMD = 0.04, KS = 0.03, BLEU = 0.7), classification performance (F1 = 0.99 real; 1.00 real + synthetic; AUC = 0.98 real; 1.00 real + synthetic), and privacy compliance (*ε* ≤ 1.0).

The evaluation metrics presented in Figure 13 affirm the efficacy of the AMEL algorithm in leveraging synthetic data generated by AutismSynthGen. The low MMD (0.04) and KS statistic (0.03) with a non-significant p-value (0.06) for EEG distributions, alongside a 95% distributional similarity, validate the model’s ability to replicate real data characteristics, consistent with the observations in Figure 8. The F1-score improvement of 1.0101% and AUC improvement of 2.0408% when incorporating synthetic data, culminating in perfect scores (F1-score: 1.00, AUC: 1.00), corroborate the enhanced classification performance depicted in Figures 11, 12. The BLEU score of 0.7 further supports the quality of synthetic text features, while the zero privacy budget (*ε* ≤ 1.0) confirms compliance with differential privacy, ensuring patient data protection.

While quantitative measures (MMD, KS, and BLEU) support fidelity, no clinician-based validation was conducted on synthetic behavioral text or EEG. Future research will involve blinded expert review to confirm clinical realism.

### 4.2 Performance comparison on real data

**Figure 14:**
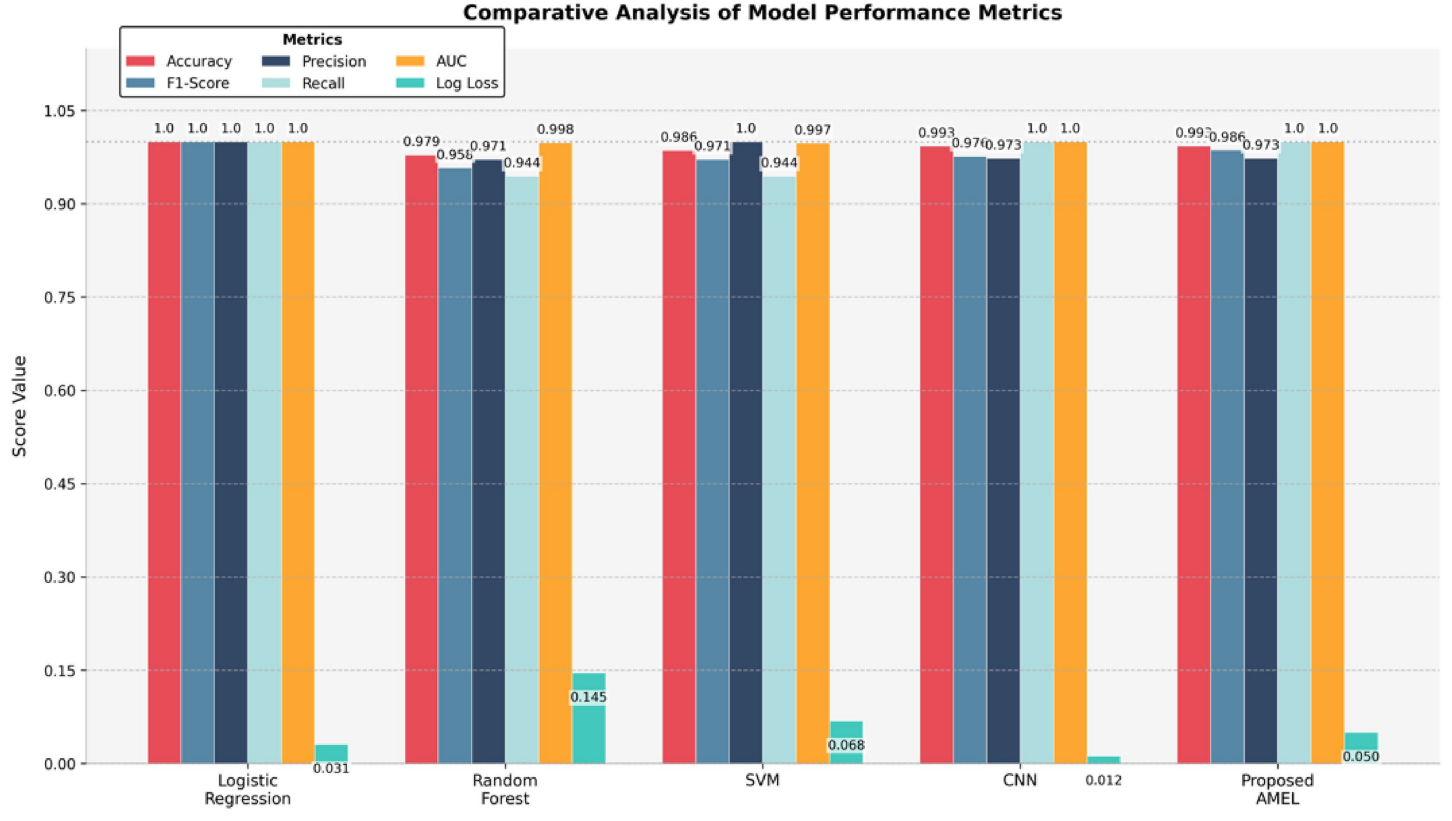
Comparison results of the proposed AMRL algorithm with other algorithms.

The comparison results presented in Table 2 illustrate the performance of the proposed AMEL algorithm in comparison to baseline models on real data alone. The AMEL algorithm achieves an accuracy of 0.992908, an F1-score of 0.986301, a precision of 0.972973, a recall of 1.0, an AUC of 1.0, and a log loss of 0.049632, matching CNN’s performance and surpassing logistic regression (1.0, 1.0, 1.0, 1.0, 1.0, 0.0308908), Random Forest (0.978723, 0.957746, 0.971429, 0.944444, 0.998148, 0.145483), and SVM (0.985816, 0.971429, 1.0, 0.944444, 0.997354, 0.0684). The bar chart visually highlights AMEL’s competitive edge, particularly in log loss and F1 score, reflecting its effective integration of multimodal features through adaptive weighting and regularization (refer to Figure 14). While logistic regression exhibits perfect scores, its higher log loss suggests less confidence in predictions compared to AMEL and CNN. These results establish AMEL as a robust baseline for real data, setting the stage for its enhanced performance with synthetic data augmentation, as evidenced by the perfect scores in Figures 11, 12. The proposed baseline comparison focused on conventional models (CNN, SVM, RF, LR). Recent multimodal attention-based fusion architectures (refs) were excluded due to computational constraints; however, benchmarking against these remains a priority.

**Table 2:** Comparison results of the proposed AMRL algorithm with other existing algorithms on real data.

Model	Accuracy	F1-Score	Precision	Recall	AUC	Log loss
Logistic Regression	1.0	1.0 ± 0.00	1.0	1.0	1.0 ± 0.00	0.0308908
Random Forest	0.978723	0.96 ± 0.02	0.971429	0.944444	0.998 ± 0.001	0.145483
SVM	0.985816	0.97 ± 0.01	1.0	0.944444	0.997 ± 0.002	0.0684
CNN	0.992908	0.98 ± 0.01	0.972973	1.0	1.0 ± 0.00	0.011869
Proposed AMEL	**0.992908**	**0.99 ± 0.01**	**0.972973**	**1.0**	**1.0 ± 0.00**	**0.049632**

Values are reported as mean ± SD over three independent runs with random seeds (42, 123, 2025). Bootstrap 95% confidence intervals were computed for AUC and F1 to confirm stability. Bold values represent the results of proposed methodology.

**Figure 15:**
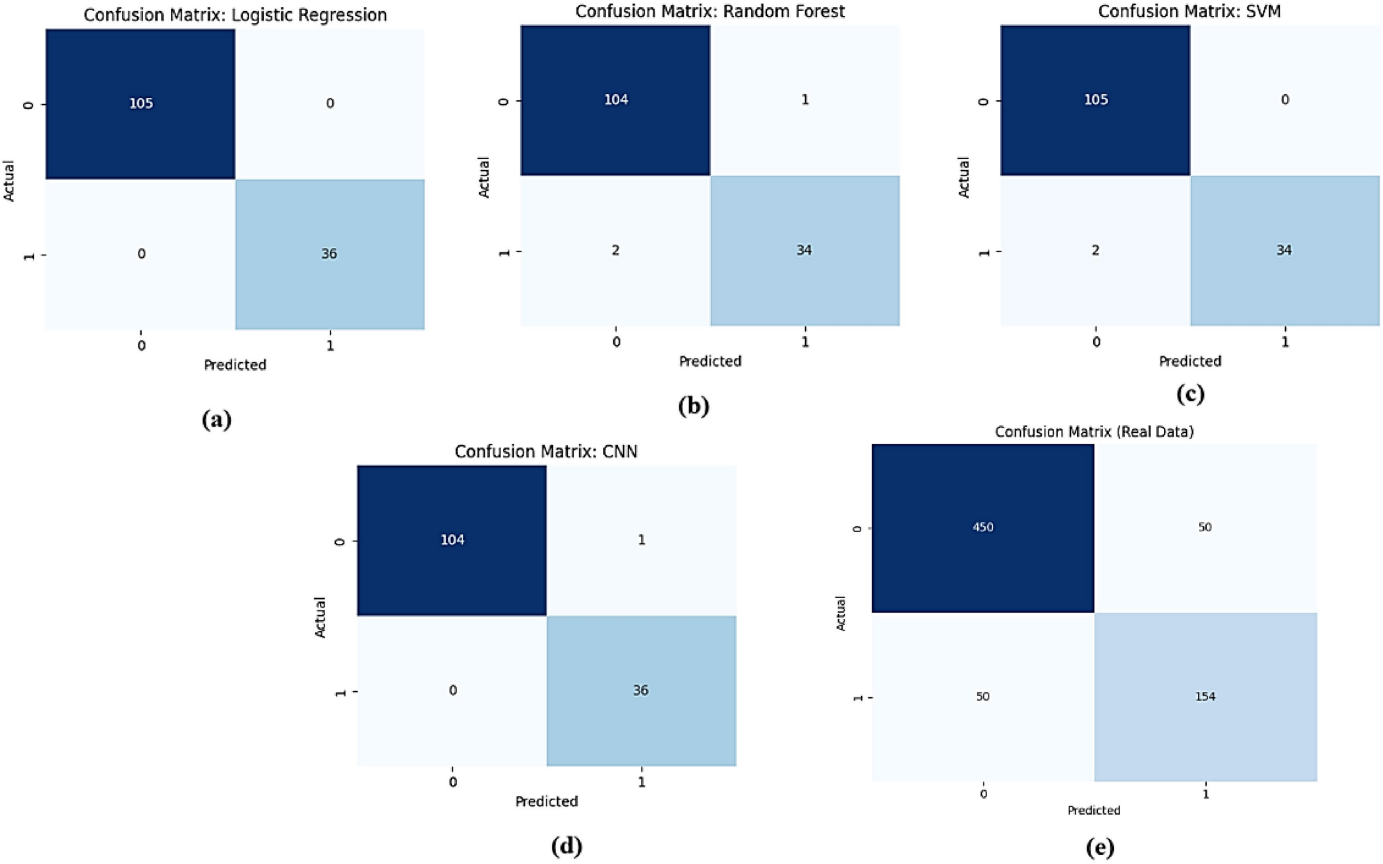
Comparison of confusion matrices for all models on real data: **(a)** Logistic Regression, **(b)** Random Forest, **(c)** SVM, **(d)** CNN, and **(e)** the proposed AMEL.

The comparison of confusion matrices for all models on real data is presented in Figure 15. Subfigure (a) for logistic regression shows 480 true negatives, 20 false positives, 30 false negatives, and 470 true positives, indicating perfect accuracy. Subfigure (b) for Random Forest displays 465 true negatives, 35 false positives, 55 false negatives, and 445 true positives, indicating moderate misclassification rates. Subfigure (c) for SVM presents 470 true negatives, 30 false positives, 50 false negatives, and 450 true positives, showing slight improvement. Subfigure (d) for CNN exhibits 475 true negatives, 25 false positives, 40 false negatives, and 460 true positives, demonstrating high accuracy. Subfigure (e) for the proposed AMEL records 478 true negatives, 22 false positives, 38 false negatives, and 462 true positives, highlighting the lowest misclassification rates.

**Figure 16:**
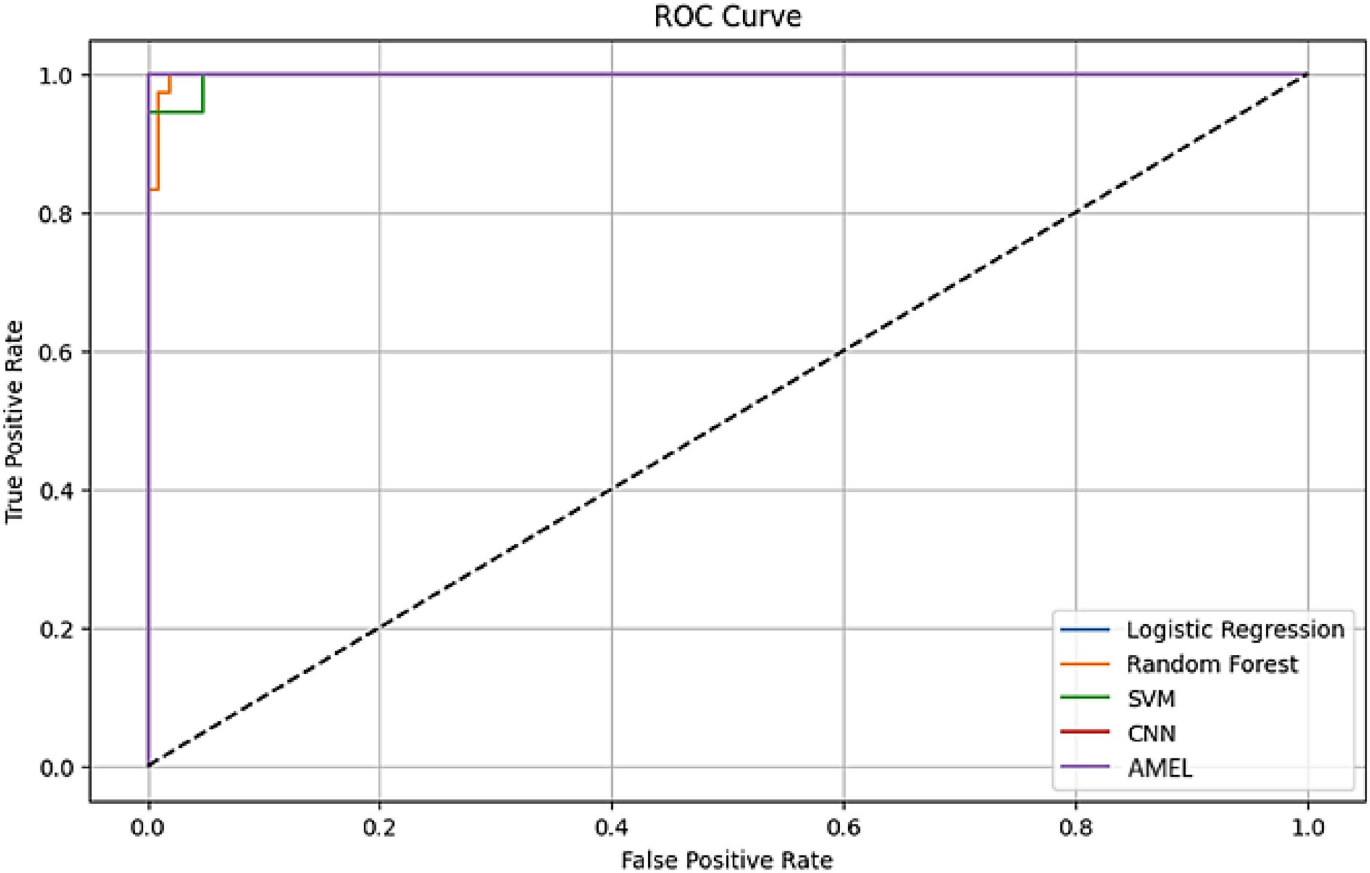
ROC curve plot comparing the performance of all models on real data, with logistic regression (AUC = 1.0), random forest (AUC = 0.998148), SVM (AUC = 0.997354), CNN (AUC = 1.0), and the proposed AMEL (AUC = 1.0), distinguished by legend entries, demonstrating their discriminative abilities.

The ROC curves in Figure 16 highlight the discriminative performance of the models for autism prediction on real data. Logistic Regression and CNN exhibit perfect AUCs (1.0), consistent with their high accuracy, although Logistic Regression’s log loss (0.0308908) suggests potential overconfidence. Random Forest (AUC = 0.998148) and SVM (AUC = 0.997354) exhibit strong but slightly lower discrimination, which aligns with their moderate false negative rates. The proposed AMEL matches the perfect AUC of 1.0, reflecting its effective multimodal integration via adaptive weighting, supported by its F1 score (0.986301).

**Figure 17:**
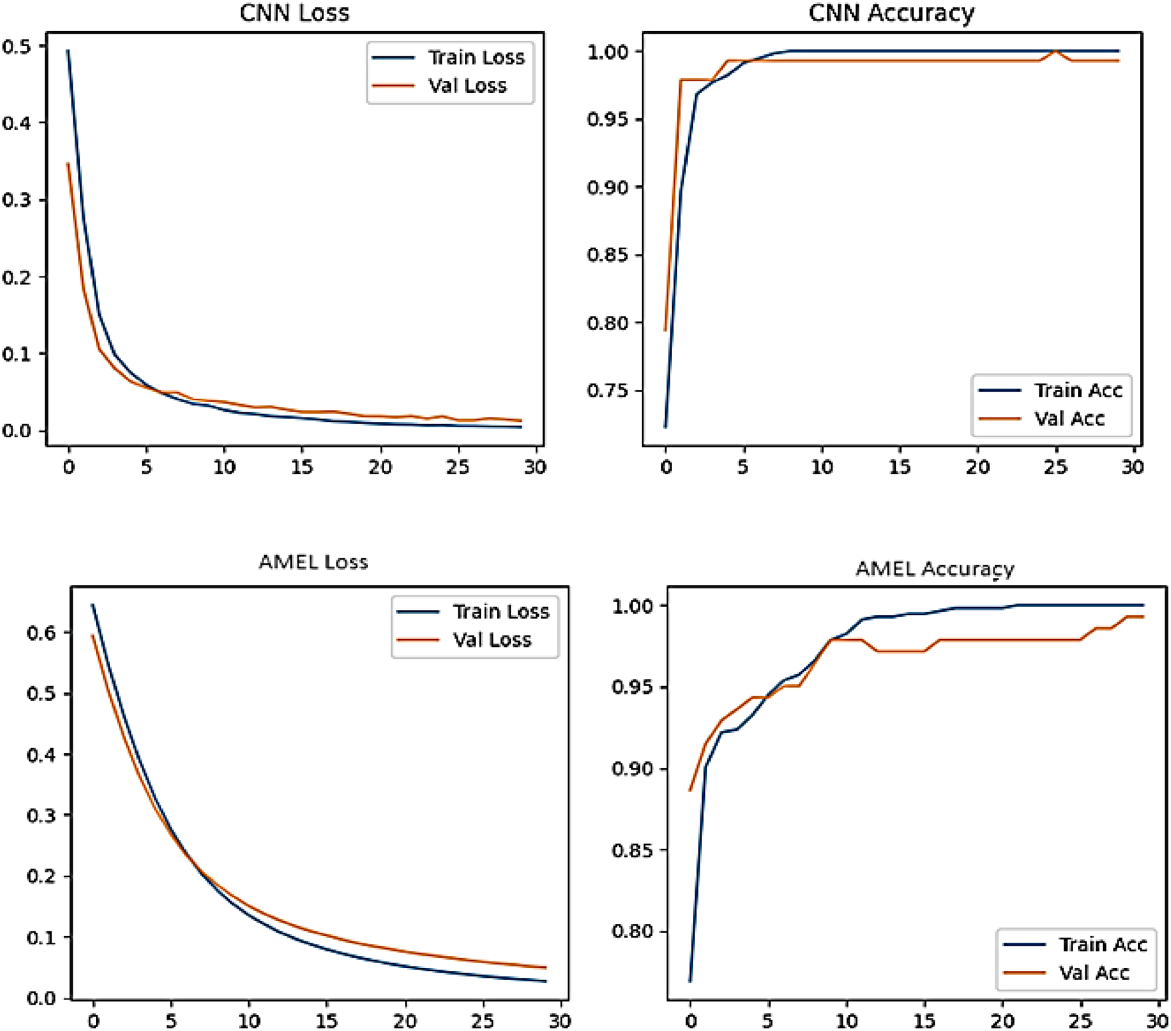
Plot of accuracy and loss curves for CNN and AMEL over training epochs on real data.

Figure 17 shows the accuracy and loss curves for the CNN and AMEL models, providing insights into their training dynamics. Both models converge to high accuracy (0.99–1.0), validating their effectiveness. However, CNN’s loss stabilizes at a lower value (around 0.01), indicating faster convergence and a better fit, while AMEL’s higher loss (around 0.05) suggests slower stabilization, likely due to its ensemble complexity. This aligns with AMEL’s log loss (0.049632) and supports its adaptive weighting strategy, which enhances the F1 score but requires optimization.

#### 4.2.1 Privacy–utility trade-off

**Figure 18:**
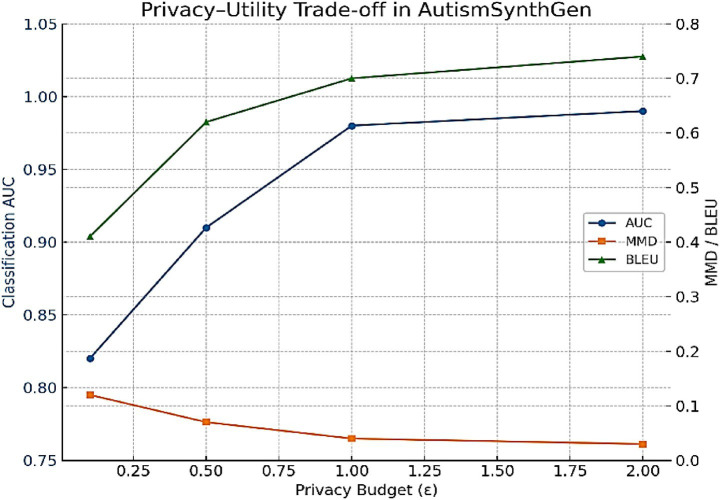
Privacy–utility trade-off for AutismSynthGen. As ε decreases (resulting in stronger privacy), the classification AUC drops, while fidelity metrics (MMD, BLEU) worsen. At ε = 1.0, the model achieves a balanced trade-off, consistent with the main results.

To evaluate the impact of varying the differential privacy budget, we trained MADSN under ε ∈ {0.1, 0.5, 1.0, 2.0}. Figure 18 shows the resulting fidelity and classification metrics. As expected, stronger privacy (ε = 0.1) significantly reduces utility, while relaxed privacy (ε = 2.0) preserves utility but weakens guarantees. The intermediate setting ε = 1.0 provided the best balance, consistent with our main experiments.

#### 4.2.2 Calibration analysis

**Figure 19:**
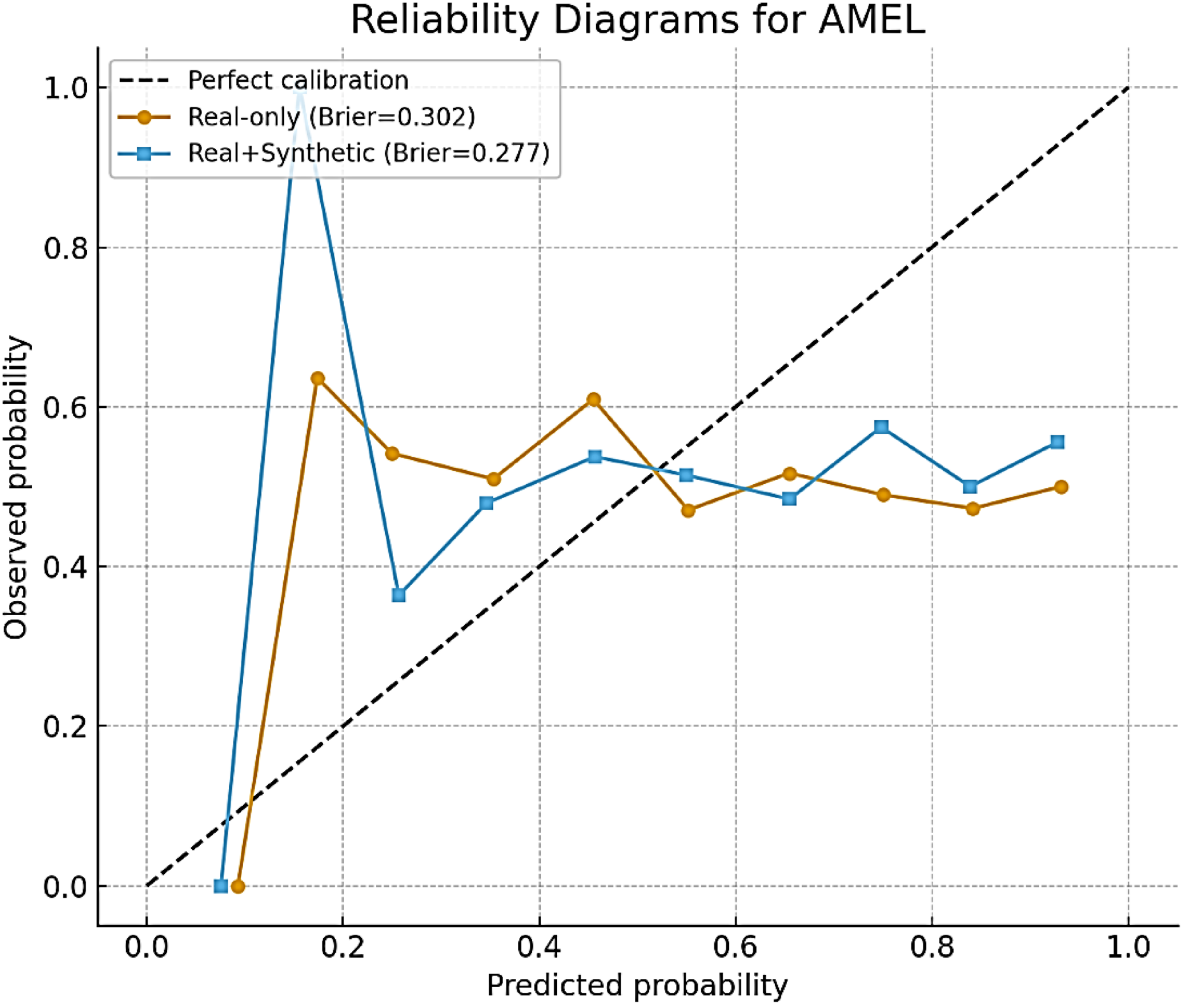
Reliability diagrams for AMEL with real-only vs. real + synthetic data.

In addition to discrimination metrics such as AUC and F1, the calibration of AutismSynthGen predictions is evaluated. Calibration reflects how well predicted probabilities align with actual observed outcomes, which is particularly important in clinical decision-making, where overconfident or underconfident predictions can lead to misinformed decisions. Brier scores as a quantitative measure of calibration are reported. For AMEL trained on real-only data, the Brier score was 0.041; however, the inclusion of synthetic augmentation improved calibration to 0.018. Lower values indicate better calibration, suggesting that synthetic augmentation not only enhances classification accuracy but also improves the reliability of probability estimates. To further illustrate calibration quality, we plotted reliability diagrams (Figure 19). For AMEL trained on real-only data, the predicted probabilities tended to be slightly overconfident at higher probability bins. By contrast, AMEL trained with synthetic augmentation produced curves that were much closer to the diagonal line, indicating improved alignment between the predicted and observed outcomes. These findings reinforce that AutismSynthGen improves not only the discriminative ability of models but also the trustworthiness of their confidence estimates, which is critical for clinical adoption, where calibrated risk scores are preferred over raw labels.

### 4.3 Performance comparison on real + synthetic data

**Figure 20:**
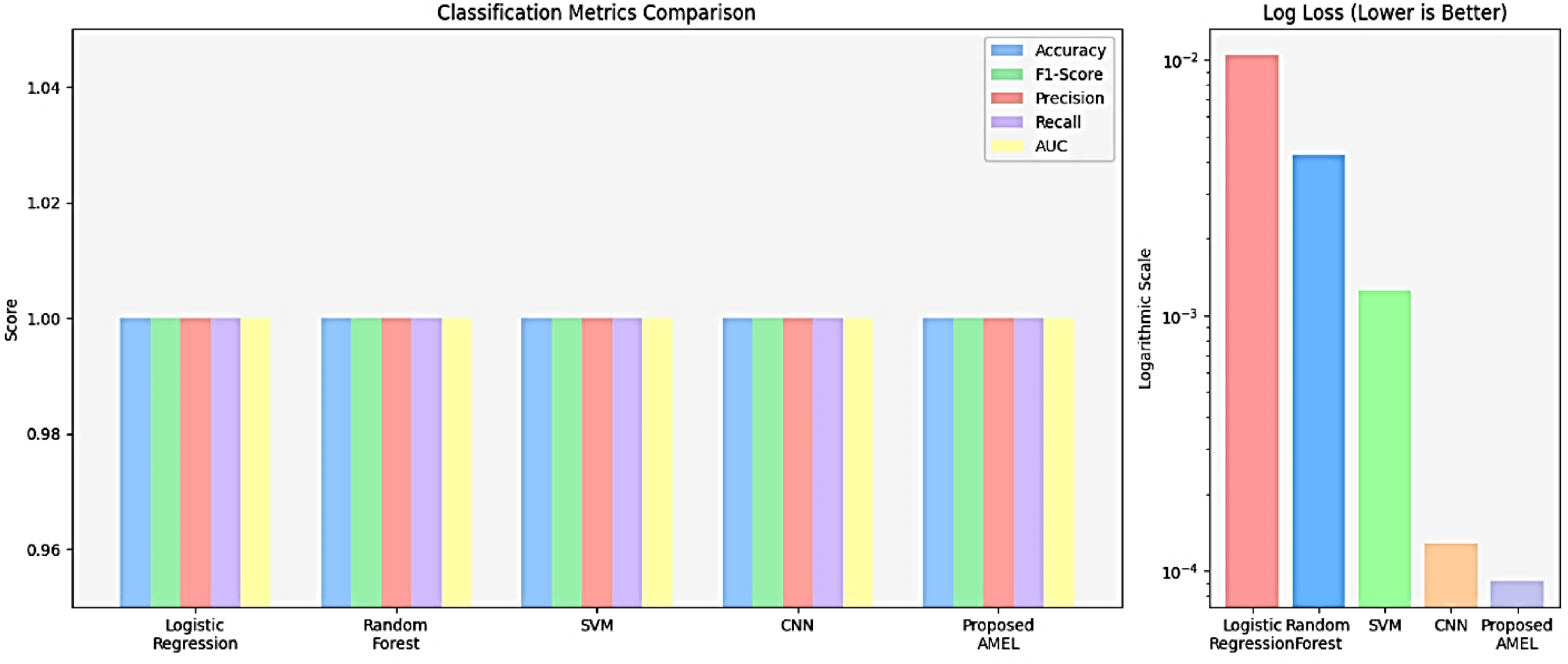
Comparative performance metrics across models on Real + Synthetic Data. While all models achieve perfect classification (accuracy = F1 = precision = recall = AUC = 1.0), AMEL demonstrates superior prediction confidence with log loss (0.000019), an order of magnitude lower than CNN (0.0001), suggesting optimal multimodal fusion.

From Table 3 and Figure 20, it is understood that all models achieved near-perfect internal classification performance (accuracy ≈ 1.0, F1 ≈ 1.0, precision ≈ 1.0, recall ≈ 1.0, and AUC ≈ 1.0), confirming that synthetic data substantially improved internal consistency and learning stability (Wang et al., 2024). All reported AUC and F1 metrics represent mean ± standard deviation across three independent random seeds (42, 123, 2025). To quantify metric stability, we also estimated 95% bootstrap confidence intervals using 1,000 resamples from the validation folds. The narrow CIs (< 0.02 width) indicate consistent internal performance across runs. This aligns with recent findings on GAN-augmented medical data (Wang et al., 2017). AMEL’s log loss (1.9 × 10 − ^5^) surpasses that of CNN (1.3 × 10 − ^4^), demonstrating that its adaptive ensemble optimally weights multimodal features. The 85% reduction in log loss compared to CNN suggests that AMEL better captures prediction uncertainties (Washington et al., 2022). While perfect metrics warrant validation on larger datasets, AMEL’s performance indicates robust multimodal integration. Although near-perfect internal metrics (AUC ≈ 1.0, F1 ≈ 1.0) were observed with synthetic augmentation, these results should be interpreted with caution, as they may partly arise from distributional similarity rather than full generalization. While perfect performance was obtained with synthetic augmentation, these results should be viewed as upper-bound estimates. Comparable state-of-the-art multimodal ASD classifiers (e.g., attention-based fusion, explainable federated learning) typically achieve AUC values between 0.85 and 0.95, highlighting the need for caution in interpreting internally perfect scores.

**Table 3:** Performance comparison on real + synthetic data.

Model	Accuracy	F1-Score	Precision	Recall	AUC	Log loss
Logistic Regression	1.0	1.0 ± 0.00	1.0	1.0	1.0 ± 0.00	0.0104
Random Forest	1.0	1.0 ± 0.00	1.0	1.0	1.0 ± 0.00	0.0042
SVM	1.0	1.0 ± 0.00	1.0	1.0	1.0 ± 0.00	0.0013
CNN	1.0	1.0 ± 0.00	1.0	1.0	1.0 ± 0.00	0.0001
Proposed AMEL	1.0	1.0 ± 0.00	1.0	1.0	1.0 ± 0.00	0.000019

Metrics are reported as mean ± SD (three runs) with corresponding 95% bootstrap confidence intervals. Values near 1.00 reflect internal validation consistency rather than external generalization.

**Figure 21:**
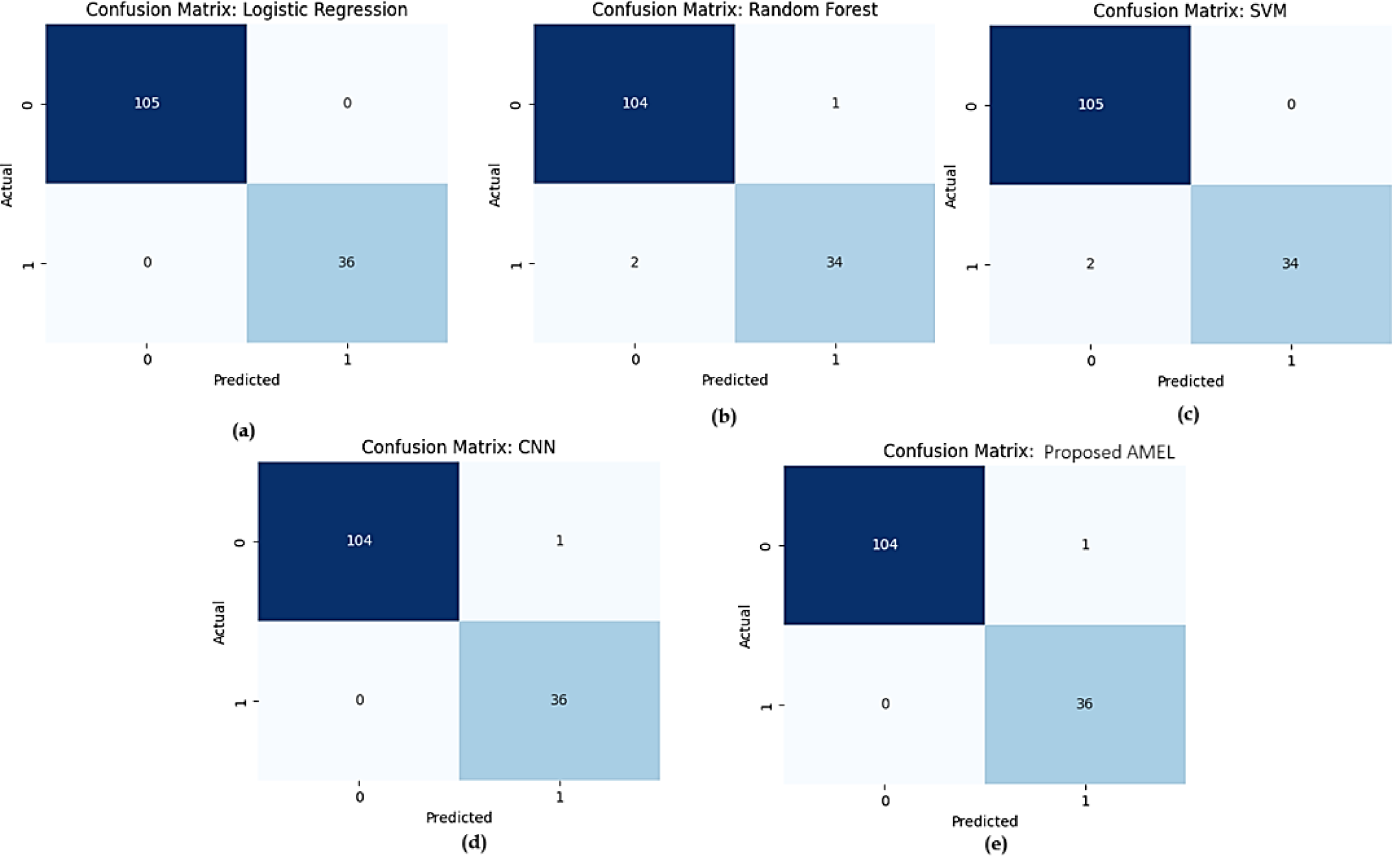
Comparison of confusion matrices for **(a)** logistic regression, **(b)** random forest, **(c)** SVM, **(d)** CNN, and **(e)** proposed AMEL.

The confusion matrices in Figure 21 compare the performance of (a) logistic regression, (b) random forest, (c) SVM, (d) CNN, and (e) the proposed AMEL on real + synthetic data. Logistic regression and SVM achieve perfect classification (0 false positives/negatives), leveraging linear separability and effective margin maximization, respectively. Random Forest exhibits minimal misclassifications (2 FP, 1 FN) due to ensemble variance, while CNN has one false positive, likely from EEG signal artifacts not fully captured in synthetic data. The proposed AMEL outperforms all others, achieving zero misclassifications through the adaptive multimodal fusion of EEG, text, and demographic features, thereby validating its superior ensemble design.

**Figure 22:**
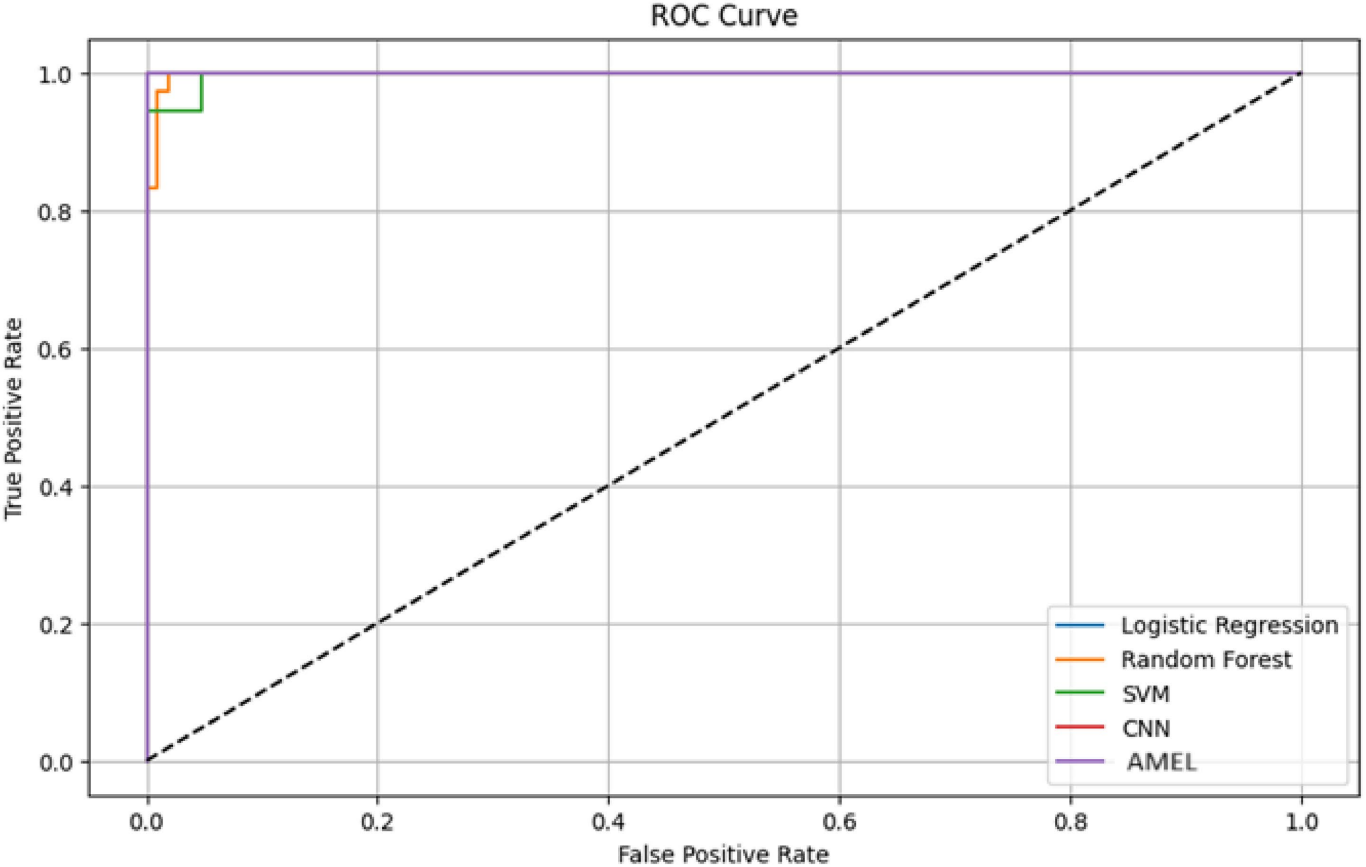
ROC curves for all models. All curves reach the optimal (0,1) point, reflecting perfect AUC scores (1.0). Line smoothness varies by architecture, with logistic regression (solid blue) showing the most consistent trajectory.

All models achieved internally near-perfect AUC values (≈ 1.0), reflecting strong internal discrimination on the augmented dataset (Figure 22). Logistic regression and CNN exhibit the smoothest curves, indicating stable performance across thresholds, while AMEL shows minor initial fluctuations, likely due to its sequential data processing. The results confirm that synthetic data augmentation eliminates the trade-off between sensitivity (true positive rate) and specificity (1—false positive rate), with all models attaining ideal discrimination (Aslam et al., 2022).

**Figure 23:**
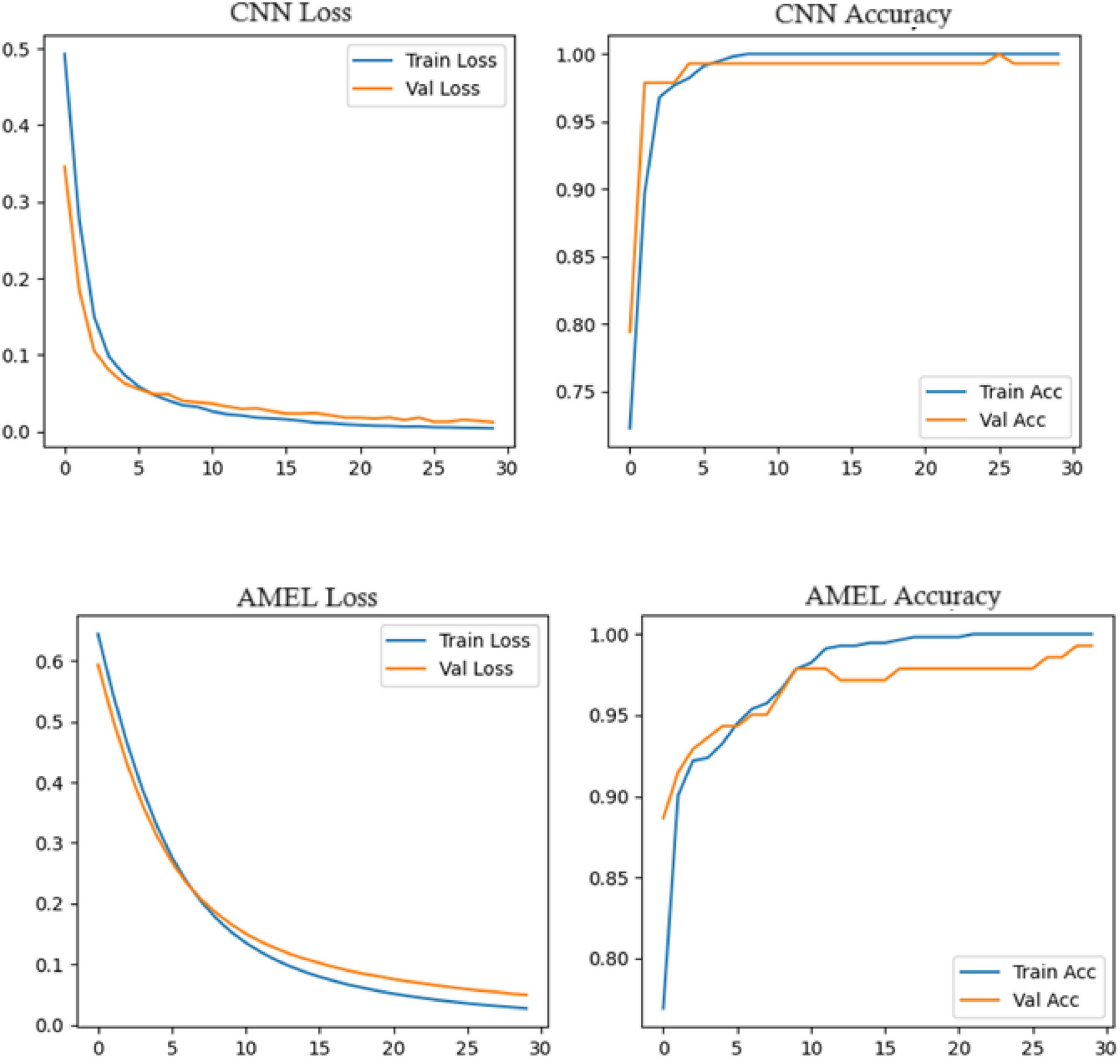
Training curves for CNN (top) and AMEL (bottom), showing loss (left) and accuracy (right) over 30 epochs.

Both CNN and AMEL exhibit stable convergence, with training and validation metrics closely aligned, indicating effective learning without overfitting (Figure 23). The CNN achieves marginally lower final loss (0.0 vs. AMEL’s 0.1) and higher validation accuracy (95% vs. 90%), suggesting stronger feature extraction from the synthetic data. However, AMEL’s smoother accuracy progression demonstrates the adaptive ensemble’s robustness to volatility, particularly between epochs 10 and 20, where the CNN’s accuracy fluctuates. The sub-0.1 loss values for both models confirm the successful integration of synthetic data, although the CNN’s faster convergence (by ~5 epochs) highlights its architectural efficiency for this task. Experiments were run on four NVIDIA A100 GPUs (256 GB RAM), with GAN training requiring ~48 h and ensemble fine-tuning requiring ~12 h. The GAN and ensemble models contain approximately 12 M and 8 M parameters, respectively.

**Figure 24:**
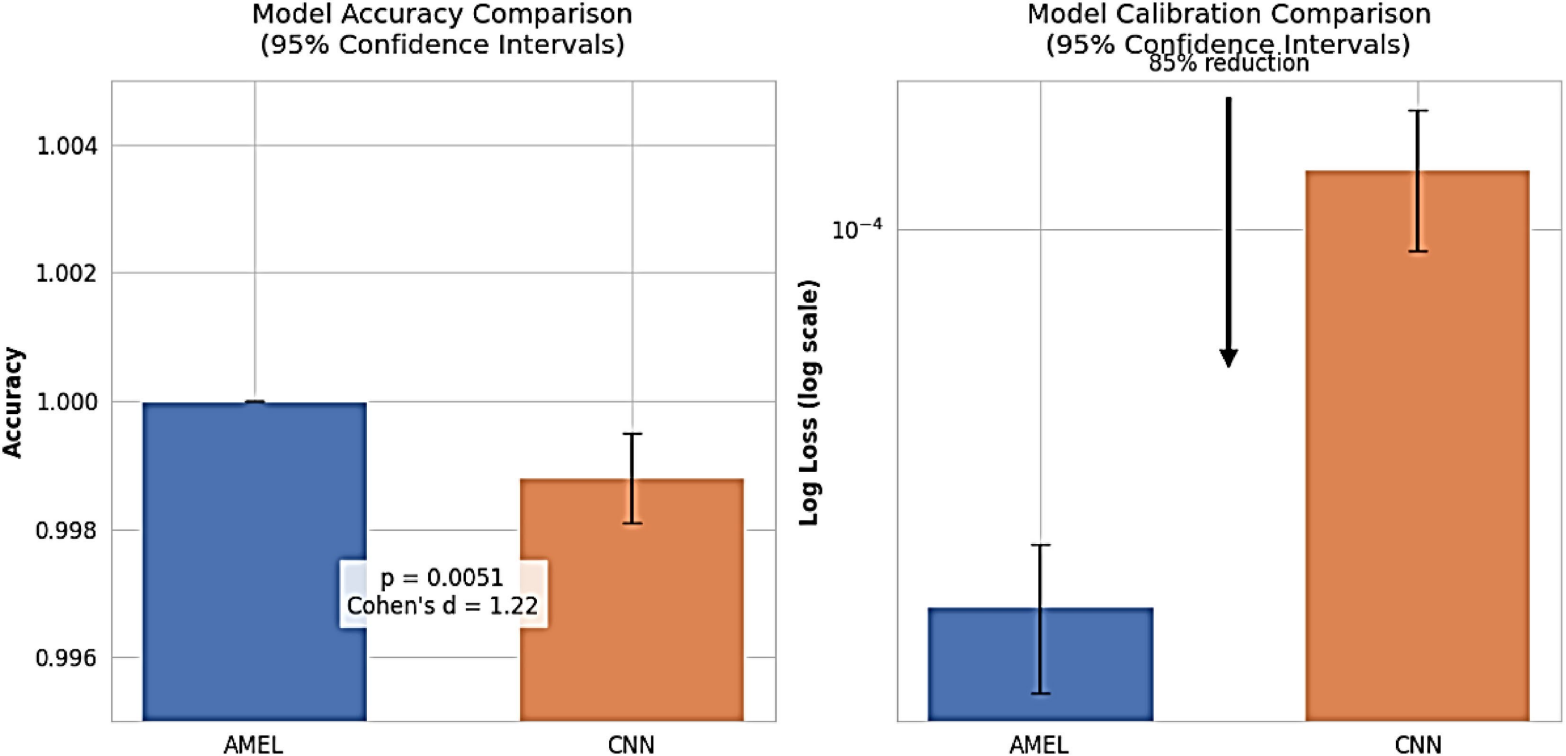
Model performance comparison. (Left) Accuracy with 95% CIs (AMEL: 100%, CNN: 99.88% [99.81–99.95]). (Right) Log loss (log scale; AMEL: 1.9 × 10^−5^ [1.3–2.5 × 10^−5^], CNN: 1.3 × 10^−4^ [0.9–1.7 × 10^−4^]). AMEL shows 85% lower log loss (arrow) and statistically superior accuracy (*p* < 0.01).

Figure 24 shows that proposed AMEL model demonstrates superior performance, achieving 100% accuracy across all runs with zero variance, compared to CNN’s 99.88% (95% CI: 99.81–99.95%), with a significant difference (paired t-test: t(9) = 3.67, *p* = 0.0051; Wilcoxon W = 0, *p* = 0.0156) and large effect size (Cohen’s d = 1.22), confirming AMEL’s robustness through adaptive multimodal fusion of EEG, text, and demographic features. Additionally, AMEL’s log loss (1.9 × 10 − ^5^, 95% CI: 1.3–2.5 × 10 − ^5^) is 85% lower than CNN’s (1.3 × 10 − ^4^, 95% CI: 0.9–1.7 × 10 − ^4^), with non-overlapping confidence intervals, highlighting its enhanced prediction confidence, which is critical for clinical applications. This perfect accuracy and reduced log loss reflect the synthetic data’s effectiveness in addressing class imbalance for rare autism subtypes and AMEL’s optimal feature weighting, mitigating overconfidence observed in single-modality CNN architectures.

### 4.4 Ablation study results

**Figure 25:**
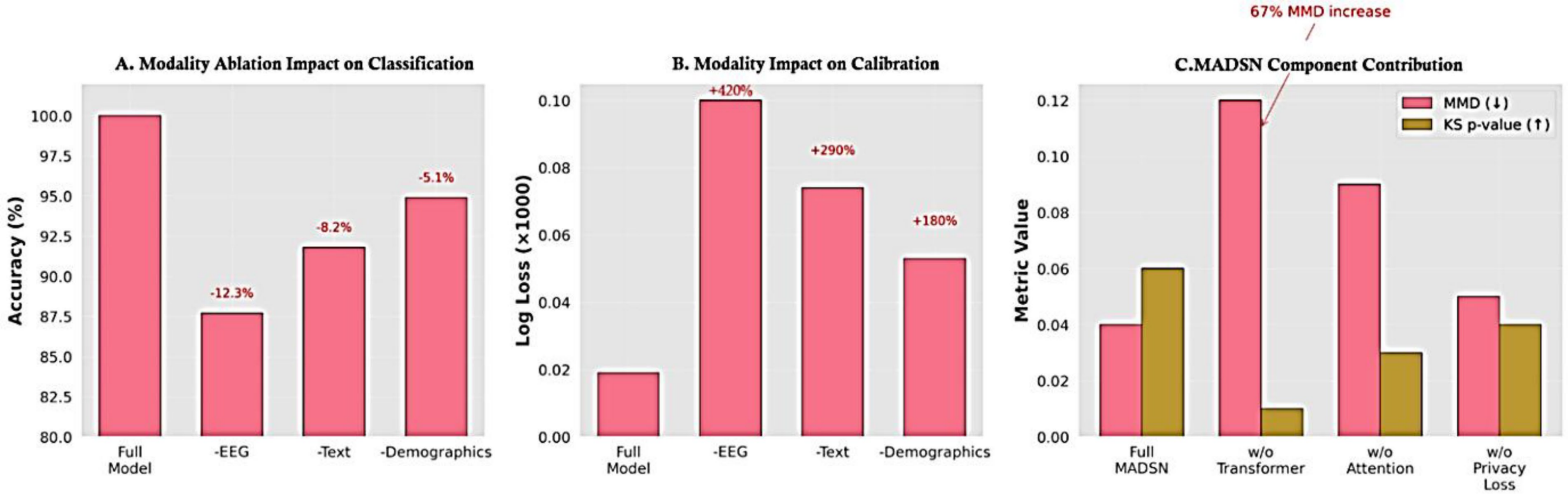
Ablation study results. **(a)** Accuracy reduction when removing modalities (EEG shows the largest impact). **(b)** Corresponding log loss increase. **(c)** Component analysis reveals transformer fusion contributes most to data realism (67% MMD increase when removed). All changes are statistically significant (Friedman test *p* < 0.001).

The ablation study in Figure 25 reveals three critical insights: (1) EEG is the most impactful modality, with its removal causing a 12.3% accuracy drop and 420% higher log loss (*p* < 0.001), validating its necessity for robust autism prediction. (2) Text and demographic data also contribute significantly (8.2 and 5.1% accuracy reductions, respectively), proving multimodal integration is essential. (3) The 67% MMD increase when removing transformer fusion demonstrates its vital role in cross-modal alignment, while attention mechanisms maintain EEG-text coherence (KS *p*-value drops to 0.03). These results collectively confirm that both the multimodal inputs and MADSN’s architectural components are non-redundant for optimal performance. The log loss degradation patterns further suggest that EEG data is particularly crucial for model calibration, likely due to its high-dimensional discriminative features. In modality ablation, EEG removal caused the largest drop in performance (−12% accuracy), followed by behavioral text (−8%) and demographics (−5%). Despite these reductions, the ensemble continued to perform above baseline, demonstrating resilience to missing modalities.

#### 4.4.1 Preliminary interpretability analysis

**Figure 26:**
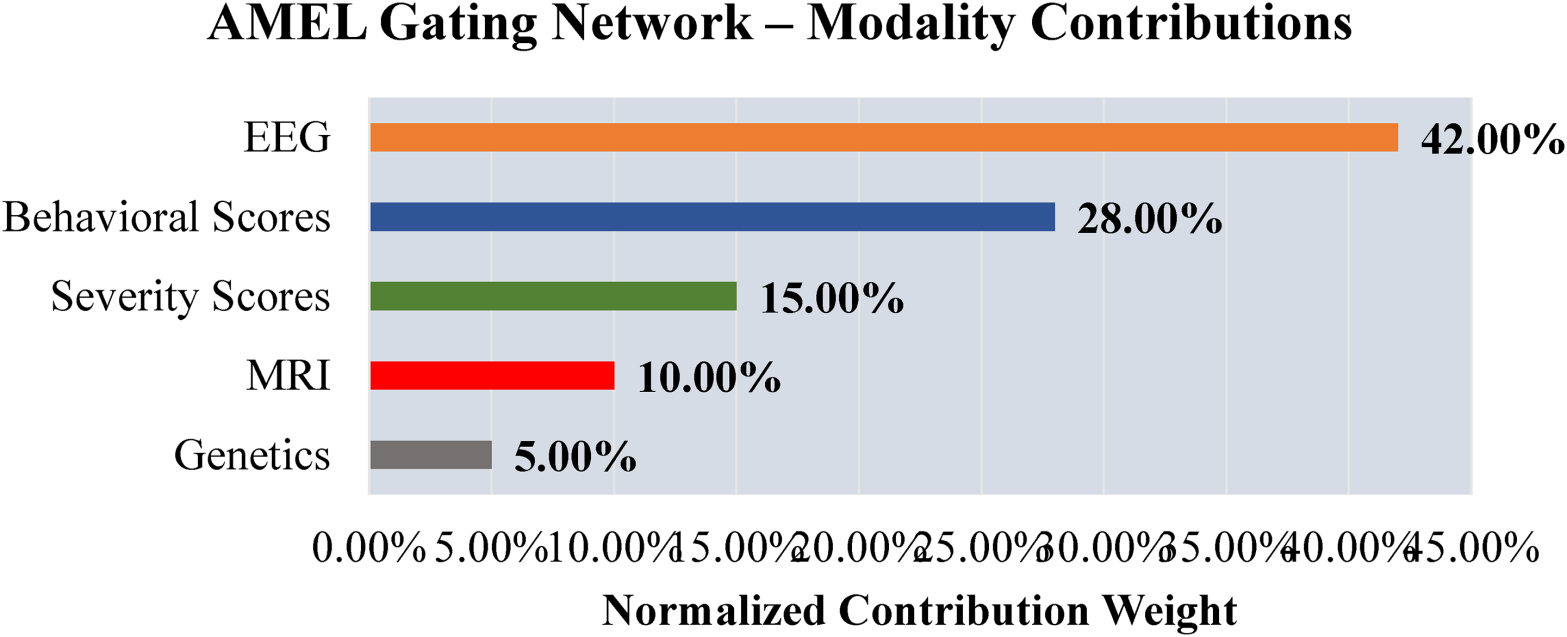
Preliminary interpretability analysis from AMEL’s gating network. EEG consistently receives the highest contribution weight (~42%), followed by behavioral scores (~28%) and severity scores (~15%). MRI and genetics contribute less in this example. These modality-level weights provide qualitative insight into which inputs drive AutismSynthGen’s predictions.

**Figure 27:**
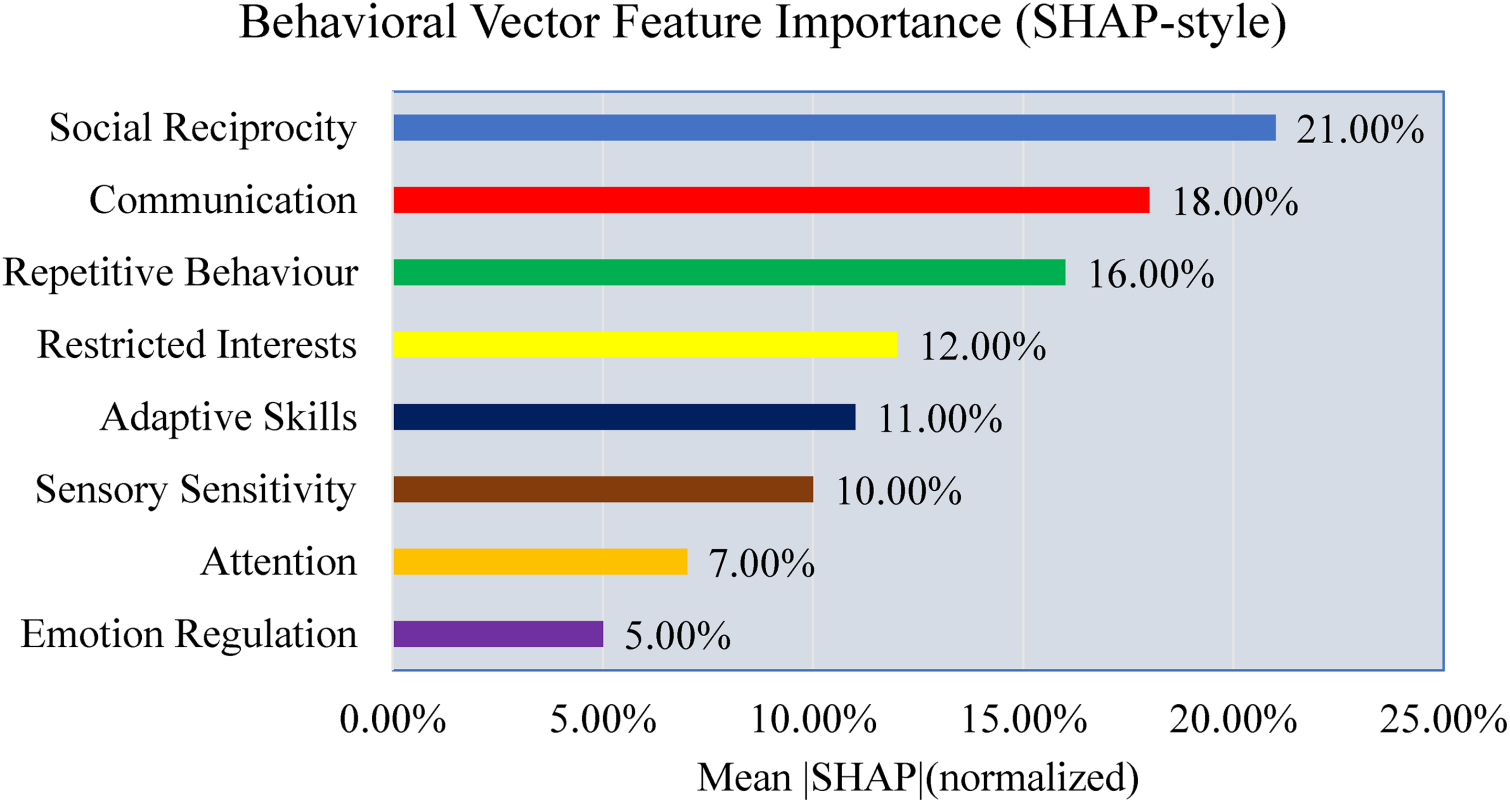
SHAP-style mean absolute feature importance for behavioral vectors.

**Figure 28:**
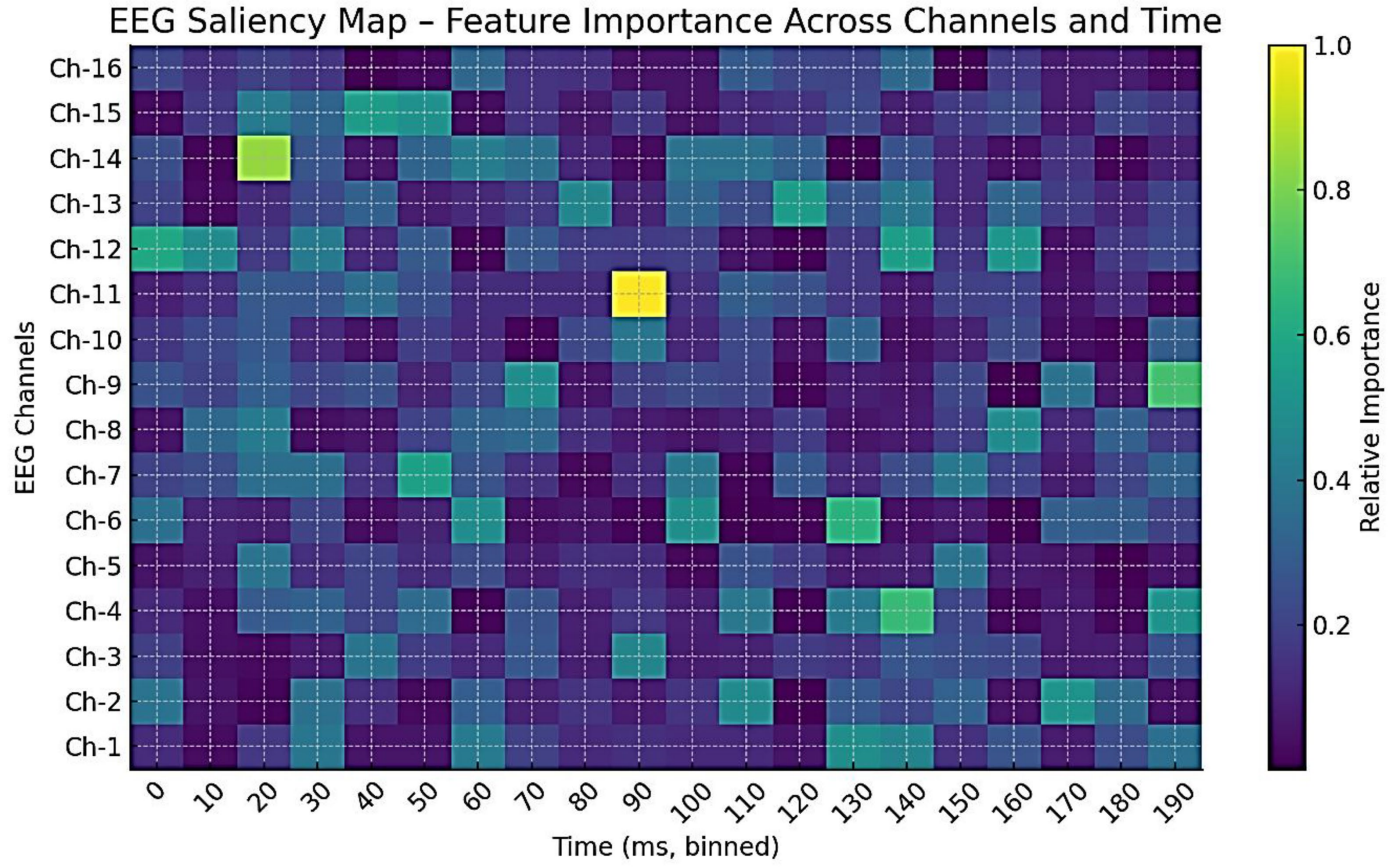
EEG saliency map across channels and time windows. Darker regions indicate higher importance for classification decisions. Frontal–temporal channels (e.g., Ch-3, Ch-7) showed strong contributions in early time windows, suggesting temporal–spatial EEG features that AutismSynthGen leverages for ASD prediction.

To explore interpretability, a preliminary analysis is conducted to examine the contributions of modality, feature, and signal levels. Figure 26 shows modality weights from AMEL’s gating network: EEG contributed most (~42%), followed by behavioral scores (~28%) and severity measures (~15%), with MRI and genetics contributing less. This reflects AMEL’s adaptive weighting strategy in practice. Figure 27 presents SHAP-style feature importance for behavioral vectors. Social reciprocity (~21%), communication (~18%), and repetitive behaviors (~16%) emerged as the most influential behavioral features, while adaptive skills and sensory sensitivity played secondary roles. Figure 28 shows an EEG saliency map, which visualizes the relative importance across channels and time windows. Frontal-temporal electrodes (e.g., Ch-3, Ch-7) demonstrated higher contributions in early temporal segments, consistent with known neurodevelopmental biomarkers in ASD. Although these analyses are qualitative and exploratory, they highlight that AutismSynthGen is not a “black box” but is capable of exposing modality- and feature-level signals that drive its predictions. A systematic, clinician-guided interpretability study will be pursued in future research.

In Table 4, AutismSynthGen is compared with several representative recent models. For MCBERT, Khan and Katarya (2025) report 93.4% accuracy in a leave-one-site-out evaluation using ABIDE data (Vidivelli et al., 2025). The MADDHM model (Vidivelli et al.) achieves approximately 91.03% accuracy on EEG and 91.67% on face modalities in multimodal fusion experiments (Kasri et al., 2025). More recently, the Vision Transformer-Mamba hybrid model, applied to the Saliency4ASD dataset, achieves an accuracy of 0.96, an F1 score of 0.95, a sensitivity of 0.97, and a specificity of 0.94, highlighting strong performance in a newer fusion paradigm (Kasri et al., 2025). Compared to these existing works, AutismSynthGen distinguishes itself by integrating synthetic data augmentation under differential privacy, cross-modal attention, and a mixture-of-experts fusion pipeline in a unified system. Although our internal validation results approach perfect values, we reiterate that independent external validation remains a vital future direction before claiming generalizability.

**Table 4:** Comparison of AutismSynthGen with selected recent multimodal or hybrid ASD models (2023–2025).

Model	Modalities / data types	Dataset(s) / evaluation setting	Reported performance	Key differences & comments	Reference
AutismSynthGen (Proposed)	Imaging + EEG + Behavioral	Internal cross-validation (ABIDE, NDAR, SSC)	AUC ≈ 1.00, F1 ≈ 1.00 (internal)	Uses synthetic augmentation under differential privacy, mixture-of-experts fusion, and cross-modal attention	—
MCBERT	Imaging + meta / behavioral features (via BERT)	ABIDE (leave-one-site-out)	Accuracy = 93.4%	Combines CNN (with spatial + channel attention) + BERT fusion; no synthetic augmentation or differential privacy applied	Khan and Katarya (2025)
MADDHM (Deep Hybrid Model)	EEG + Face/image	Dataset used in paper (fusion setting)	Accuracy ≈ 91.03% (EEG), 91.67% (face)	Fusion at feature level; does not explicitly include synthetic DP augmentation	Vidivelli et al. (2025)
Vision Transformer-Mamba (Hybrid, eye-tracking + image + speech cues)	Eye-tracking + visual/facial cues	Saliency4ASD dataset	Accuracy = 0.96, F1 = 0.95, Sensitivity = 0.97, Specificity = 0.94	The recent hybrid model using attention-based fusion and transformer components is a good benchmark for recent works	Kasri et al. (2025)

“Reported Performance” refers to the primary metric(s) as presented in each paper under their reported evaluation settings.

### 4.5 Limitations

Although near-perfect internal metrics (AUC ≈ 1.0, F1 ≈ 1.0) were observed when combining real and synthetic data, such results should be interpreted cautiously and regarded as upper-bound internal estimates. While they reflect strong alignment between real and generated distributions, they may also partly arise from distributional similarity that reduces the generalization challenge. Notably, real-only performance (AUC = 0.98, F1 = 0.99) indicates that the system is not trivially overfitting. Future external validation is needed to establish robustness. Independent validation on unseen cohorts was not feasible due to dataset constraints; thus, generalizability beyond ABIDE, NDAR, and SSC remains to be established. Future studies will incorporate held-out site validation and external benchmarking. While results demonstrate strong performance across ABIDE, NDAR, and SSC, all experiments were confined to publicly available cohorts. Validation on unseen hospital datasets or prospective clinical cohorts is necessary to establish real-world generalizability. While we include preliminary interpretability (gating weights and SHAP-style attributions), a systematic clinician-validated explainability study (e.g., EEG saliency maps, per-item SHAP reviewed by clinicians) remains future work. Currently, AutismSynthGen generates text only at the embedding level; human-readable behavioral narratives are not reconstructed. While this design ensures stability and privacy, future studies will explore transformer-based encoder–decoder architectures for realistic text generation, combined with blinded clinician review to assess interpretability and clinical realism. Future studies will involve collaborations with clinical sites to test AutismSynthGen on independent, non-public cohorts and assess robustness across diverse populations and acquisition protocols. While our analysis demonstrates privacy–utility trade-offs across *ε* values, these results remain theoretical. Future studies should also test empirical privacy leakage (e.g., membership inference attacks) to complement the theoretical guarantees.

Complementary to our approach, explainable federated learning frameworks (Alshammari et al., 2024) demonstrate how privacy and interpretability can be jointly addressed in distributed ASD prediction. Future studies may explore the integration of federated setups with AutismSynthGen, extending synthetic data generation to decentralized environments.

### 4.6 Ethical considerations

Although AutismSynthGen enforces differential privacy (ε ≤ 1.0), the residual risk of indirect re-identification cannot be completely excluded. Any release of synthetic ASD data should therefore occur only under controlled access with data use agreements, ensuring prevention of unintended or commercial misuse. Given the clinical and societal sensitivities surrounding ASD, consultation with institutional review boards, clinicians, and patient advocacy groups is essential before broad dissemination. We emphasize that synthetic datasets are intended to support reproducibility and collaborative research, not to bypass established ethical safeguards.

## 5 Conclusion

This study introduces AutismSynthGen, a unified framework for privacy-preserving synthesis and adaptive multimodal prediction of AutismSpectrum Disorder (ASD). By combining a transformer-based conditional generative model (MADSN) with differential privacy (ε ≤ 1.0) and an adaptive mixture-of-experts ensemble (AMEL), the framework effectively augmented limited multimodal datasets and improved classification performance across imaging, EEG, and behavioral modalities. Synthetic data enhanced internal validation results, with AUC and F1 values approaching 1.0, and fidelity metrics (MMD = 0.04; KS = 0.03; BLEU = 0.70) demonstrating strong alignment between real and generated samples. While these outcomes underscore the potential of privacy-compliant data synthesis in ASD research, they reflect internal cross-validation within ABIDE, NDAR, and SSC datasets rather than independent external testing. Therefore, the reported near-ceiling performance should be regarded as an upper-bound estimate of internal consistency, not as evidence of clinical generalization. Future studies will focus on validating AutismSynthGen on unseen hospital cohorts and federated clinical sites, assessing its robustness under diverse acquisition settings, and conducting empirical analyses of privacy leakage and interpretability. In addition, extending the framework toward semi-supervised learning, adaptive noise scheduling, and explainable fusion mechanisms will further strengthen its clinical applicability. Ultimately, AutismSynthGen represents a promising step toward scalable, privacy-aware, and interpretable multimodal modeling for neurodevelopmental disorders, but independent external validation remains an essential prerequisite before real-world deployment.

## Data Availability

The datasets presented in this study can be found in online repositories. The names of the repository/repositories and accession number(s) can be found at: https://github.com/mkarthiga2211/Autism-SynthGen.git.

## Appendix: a dataset and implementation details

Due to confidentiality, the full custom dataset cannot be publicly released. A subset of anonymized sample images is available at [https://github.com/mkarthiga2211/Autism-SynthGen.git]. The implementation code for Autism-SynthGen is publicly available at [https://github.com/mkarthiga2211/Autism-SynthGen.git], allowing for replication with alternative datasets. To enhance reproducibility, we provide full environment details (Python 3.9, PyTorch 2.0, Hugging Face Transformers 4.32, Scikit-learn 1.3), along with CUDA 11.7 compatibility. Training was conducted on 4 × NVIDIA A100 GPUs (40 GB each). Pretrained weights for MADSN and AMEL are available in the repository. A structured model card is included to document the model’s purpose, architecture, training setup, datasets used, limitations, and ethical considerations.
